# Supplementary material for: Macrophage-Derived upd3 Cytokine Causes Impaired Glucose Homeostasis and Reduced Lifespan in Drosophila Fed a Lipid-Rich Diet
Source: Immunity. 2015 Jan 20;42(1):133–44. doi: 10.1016/j.immuni.2014.12.023 (PMC4304720; doi:10.1016/j.immuni.2014.12.023)

Immunity, Volume *57*

Supplemental Information

Macrophage-Derived *upd3* Cytokine Causes Impaired Glucose Homeostasis and Reduced Lifespan in *Drosophila* Fed a Lipid-Rich Diet

Katie J. Woodcock, Katrin Kierdorf, Clara A. Pouchelon,
Valérie Vivancos, Marc S. Dionne, and Frédéric Geissmann

Supplemental information

Supplemental Figure Legends

Figure S1, related to Figure 1.

A. Survival of adult male Oregon-R flies fed a control diet or lard diet. Data pooled from 3 independent experiments. Log-rank χ^2=56.31, p<0.0001, Wilcoxon test χ^2=43.68, p<0.0001. **B.** Survival of w^{1118} flies fed a control or lard diet at 29°C. Data pooled from 6 independent experiments. Log-rank χ^2=212.6, p<0.0001, Wilcoxon χ^2=159.2, p<0.0001. **C.** Uptake of control and lard-enriched blue labelled food over 4 hours by groups of 10, 10 day old flies, n=4, mean absorbance at 625±SEM, p=0.652 ns. **D.** Time course of *Ilp2, 3* and *5* transcript levels in flies fed a control or lard diet at days 10 and 30, 3 flies per sample, histogram represents mean± SEM of 4-5 independent samples per diet and per time point. **E.** Repeat western blots as in Figure 1.D.

Figure S2, related to Figure 2.

A. Confocal microscopy of 10XSTAT92E-GFP reporter fly leg muscle after 10 days on a control or lard diet as a supplement to Figure 2.C. **B.** Confocal microscopy images of 10XSTAT92E-GFP reporter fly guts, flies were fed a control or lard diet for 20 days prior to dissection, as a supplement to Figure 2.D. **C.** *Defensin, Attacin-A, Metchnikowin, Cecropin A1* and *Drosocin* transcript levels in unchallenged (white bars) and *E. coli* and *M. luteus* infected flies (black bars) after 30 days of control or lard enriched diets. Histograms represent mean± SEM of 4-5 independent samples per diet.

Figure S3, related to Figure 3.

Representative confocal microscopy of $Hml\Delta^+$ (white) and crq^+ (green) expression in a 10 day old fly. Bar: 50µm. Right: quantification *HmlΔ* and *crq* double positive and single positive cells, mean± SEM, n=4.

Figure S4, related to Figure 4.

A. Survival of inducible muscle (*24B*) driven *upd3* knock down flies (24BGal80^{ts}>*upd3*-IR, closed shapes) compared to control flies (UAS-*upd3*-IR/+, open shapes) on control diet and lard diet. Data pooled from 3 independent experiments. Log-rank χ^2=2.638, p=0.1043, Wilcoxon χ^2=0.3661, p=0.5452 for flies on a lard diet. **B.** Idem, for midgut (*mex1*) driven *upd3* knock down flies (*mex1>upd3*-IR, closed shapes) and control flies (UAS-*upd3*-IR/+, open shapes). Data pooled from 3 independent experiments. Log-rank χ^2=0.7216, p=0.3956, Wilcoxon χ^2=2.201, p=0.1379 for flies on a lard diet. **C.** Western blot analysis of p-AKT normalised to *Tubulin*, supplement to Figure 4D. **D-E.** Repeat western blots as in Figure 4D. **F.** Plasmatocyte numbers, ascertained by confocal microscopy of 65 day old *upd3* knock down (*Hml*Δ-Gal4,UAS-2xeGFP/*upd3*-IR, white bar) and control flies (*Hml*Δ-Gal4,UAS-2xeGFP/+, grey bar), mean± SEM, n=4.

Figure S5, related to Figure 5.

Survival of *upd3*-null rescue flies and controls fed a control diet. Data pooled from 5 independent experiments. Log-rank χ^2=44.40, p<0.0001, Wilcoxon χ^2=39.36, p<0.0001.

Figure S6, related to Figure 6.

A. Confocal images of plasmatocyte depleted and control flies injected with DiI-LDL, as a supplement to Figure 6. C. **B.** Transcript levels of *Ilp2*, *3* and *5* in plasmatocyte depleted and control flies fed a lard enriched diet for 20 days, 3 flies per sample, histogram represents mean± SEM of 4-5 independent samples per diet.

Figure S7, related to Figure 7.

A. Scavenger receptor screen summary table. Hemese, a glycophorin like membrane molecule (Kurucz et al., 2003), is a control expressed by plasmatocytes. Expression in sorted plasmatocytes and efficiency of plasmatocyte specific knock down (*HmlΔ*-Gal4) were assessed by qPCR. Knock down in the whole fly was studied using *Tub*-Gal4. **B.** Transcript level of *NimC1* and *crq* in plasmatocytes FACS sorted from *crq* and *NimC1* knock down flies and controls **C.** Survival of a second plasmatocyte-specific *crq* knock down fly line compared to controls. Data pooled from 4 independent experiments. Log-rank χ^2=114.9, p<0.0001, Wilcoxon χ^2=98.04, p<0.0001 for flies on a lard diet. **D.** Transcript levels of *Ilp2*, *3* and *5* in *crq*-IR/+ and

HmlΔ>crq-IR flies fed a lard enriched diet for 20 days, 3 flies per sample; histogram represents mean± SEM of 4-5 independent samples per diet. **E.** Survival of macrophage-specific *NimC1* knock down flies and controls. Data pooled from 3 independent experiments. Log-rank $\chi2$=0.2991, p=0.5844, Wilcoxon $\chi2$=0.1908, p=0.6623 for flies on a lard diet. **F.** Plasmatocyte numbers, ascertained by confocal microscopy of *bsk* knock down (white bar) and control flies (black bar), mean± SEM, n=4-5. **G.** *Socs36E* and *puckered (puc)* transcript levels in plasmatocyte *crq* knock down (*HmlΔ>crq*-IR, grey) and control (UAS-*crq*-IR/+, white) flies fed a control or lard diet for 30 days, 3 flies per sample, histogram represents mean± SEM of 4-5 independent samples per diet. **H.** Schematic model proposed to explain the roles of macrophages in the reduction of insulin sensitivity and lifespan due to lipid-rich diet.

Supplemental references

Hombria, J.C., Brown, S., Hader, S., and Zeidler, M.P. (2005). Characterisation of Upd2, a Drosophila JAK/STAT pathway ligand. Dev Biol 288, 420-433.

Kurucz, E., Zettervall, C.J., Sinka, R., Vilmos, P., Pivarcsi, A., Ekengren, S., Hegedus, Z., Ando, I., and Hultmark, D. (2003). Hemese, a hemocyte-specific transmembrane protein, affects the cellular immune response in Drosophila. Proc Natl Acad Sci U S A 100, 2622-2627.

Sinenko, S.A., and Mathey-Prevot, B. (2004). Increased expression of Drosophila tetraspanin, Tsp68C, suppresses the abnormal proliferation of ytr-deficient and Ras/Raf-activated hemocytes. Oncogene 23, 9120-9128.

Supplemental Experimental Procedures

Drosophila melanogaster stocks

Fly stocks were maintained on food containing 10% w/v Brewer’s yeast, 8% fructose, 2% polenta and 0.8% Agar, at 25°C and 60% relative humidity. Male flies were used for all experiments.

**Fly stock**	**Description**
w^{1118};*HmlΔ*Gal4,UAS-2xeGFP	Plasmatocyte specific reporter line, allowing for plasmatocyte visualisation (Sinenko and Mathey-Prevot, 2004). Gift of Sergey Sinenko.
w^{1118};;*HmlΔ*-DsRed	Plasmatocyte specific reporter line. Allowing for plasmatocyte visualisation in Gal4, UAS independent manner (Clark et al., 2011).
w^{1118};;*crq*-Gal4/TM6C.Sb^1	Plasmatocyte expressed driver.
w^{1118};UAS-2xeGFP;*crq*-Gal4/TM6C.Sb^1	Plasmatocyte expressed reporter line.
10X*STAT92E*-GFP flies	STAT-GFP reporter line (Bach et al., 2007)
w;;UAS-*reaper*/TM3	Overexpression line for proapoptotic protein reaper

	(*rpr*).
w^{1118};*Hml*ΔGal4,UAS-2xeGFP;Tub-Gal80^{ts}/TM6C.Sb^1	Temperature sensitive plasmatocyte specific reporter line.
wupd3 null (on the X chromosome)	Kindly donated by Bruno Lemaitre (Osman et al., 2012)
w;;UAS-*NimC1*-IR	Bloomington stock centre number: 25787
w;UAS-*crq*-IR & *w*;;UAS-crq-IR	VDRC stock numbers: 45883 (*crq*-RNAi-1) and 45884 (*crq*-RNAi-2)
w;UAS-*upd3*-IR	VDRC stock number: 106869
w;*upd3*-Gal4,UAS-GFP	Allowing for visualisation of $upd3^+$ cells
w;UAS-*Hemese*-IR	VDRC stock number: 107469
w;UAS-*CG10345*-IR	VDRC stock number: 100252
w;*mex1*-Gal4	Midgut specific driver line. Kindly donated by Nicolas Buchon.
w^{1118};UAS-*upd3*/SM6a	Line for over expression of *upd3*
w^{1118};;UAS-*upd3*/TM6C.Sb^1	Line for over expression of *upd3*
w;;UAS-*Myd88*-IR	VDRC stock number: 25399
w;UAS-*Imd*-IR	VDRC stock number: 101834
w;UAS-*Tak1*-IR	VDRC stock number: 101357
*w*UAS-*STAT92E*-IR;UAS-*STAT92E*-IR	Bloomington stock number: 26899 (on X) and VDRC stock number:106980 (on 2nd)
w;UAS-*bsk*-IR	VDRC stock number: 104569
w;UAS-*upd2*-GFP	Line for over expression of *upd2*. Kindly donated by James C G Hombria (Hombria et al., 2005)
w^{1118};;*Tubulin*-Gal4	Ubiquitous driver line
w^{1118};*Tubulin*-Gal80^{ts};*24B*-Gal4	Temperature sensitive muscle expressed driver line.
w;c564-Gal4;*Tubulin*-Gal80^{ts}	Temperature sensitive fat body specific driver line.

Quantitative Real-Time PCR

RNA extractions were carried out in TRIzol (Invitrogen) following the manufacturer's directions. cDNA synthesis was carried out using the First Strand cDNA Synthesis Kit (Thermo Scientific), priming with random hexamers (Thermo Scientific). PCR was performed with Sensimix SYBR Green no-ROX (Bioline) on a Corbett Rotor-Gene 6000. The cycling conditions used throughout were as follows: Hold 95°C for 10 minutes, then 40 cycles of 95°C for 15s, 63.4°C for 30s, 72°C for 30s. All calculated gene expression values were initially normalized to the value of the loading control gene, *Rpl1*, prior to further analysis.

The primer sequences used in this study are as follows:

Primer (qPCR)	Left	Right
Rpl1	TCCACCTTGAAGAAGGGCTA	TTGCGGATCTCCTCAGACTT
upd3	ACTGGGAGAACACCTGCAAT	GCCCGTTTGGTTCTGTAGAT
NimC1	CGCACCGAGTATTATACAGACG	TCCATGTTGAGGACACTGTTG
Ilp2	ATCCCGTGATTCCACCACAAG	GCGGTTCCGATATCGAGTTA

Ilp3	CAACGCAATGACCAAGAGAA	TGAGCATCTGAACCGAACT
Ilp5	GCCTTGATGGACATGCTGA	AGCTATCCAAATCCGCCA
Socs36E	AAAAAGCCAGCAAACCAAAA	AGGTGATGACCCATTGGAAG
crq	GCAGATAACCTTGTAGAGGATGG	CTCAGGTAAATGGGGATAGGTG
Dpt	ACCGCAGTACCCACTCAATC	CCCAAGTGCTGTCCATATCC
Drs	GTACTTGTTCGCCCTCTTCG	CTTGCACACACGACGACAG

Oil Red O staining of FACS-sorted plasmatocytes

See Supplemental Experimental Procedures.

FACS sorted plasmatocytes were stained for neutral triglyceride and lipid presence using Oil Red O (Sigma-Aldrich). Cells were sorted into 1x PBS-EDTA (2mM), after the sort cells were spun down at 4°C for 7 minutes at 150g. The cells were re-suspended in 50μl *Drosophila* Schneider's medium (Invitrogen), and were transferred onto a poly-L-lysine (Sigma-Aldrich) coated coverslip, which was placed inside a well of a 24 well non-tissue culture plate. After a 30 minute incubation at 37°C a further 500μl Schneider's medium was added and the incubation was repeated. All media was then removed and the cells were fixed with 4% PFA (paraformaldehyde) (Sigma-Aldrich) for 30 minutes at room temperature. After this the PFA was removed, and the cells were washed quickly with 60% isopropanol (Sigma-Aldrich). Oil Red O was then added to the cells for 30 minutes at room temperature. The Oil Red O was then removed and the coverslip was washed with distilled water. The coverslip was then inverted and mounted onto a superfrost slide (VWR International), in 10μl of Vectashield mounting media (Vector Laboratories) containing 1/1000 DAPI (4',6-diamidino-2-phenylindole) for nuclei staining. Mounting media was left to set for approximately 24 hours before cells were imaged with a confocal microscope (Leica SP5).

Thin layer chromatography (TLC) Triglyceride measurement

TLC experiments were adapted from (Al-Anzi and Zinn, 2010). Groups of 10 flies, in quadruplicate per genotype per diet, were anesthetised using CO_2 and then placed in 100μl of 3:1 ratio of chloroform: methanol. Samples were centrifuged for 3 minutes at 13,000rpm at 4°C, and then flies were smashed with pestles. A set of standards were prepared using lard in 3:1 chloroform;methanol for quantification. Samples and standards were loaded onto a silica gel, and a 4:1 mix of hexane: etheyl ether was prepared as the mobile phase. Gels were then stained using a general oxidising stain, ceric ammonium heptamolybdate (CAM), and baked at 80°C for 20 minutes. Baked gels were imaged with a BioRad Molecular Imager and triglyceride bands were quantified using BioRad Image Lab software.

Feeding assay

Blue diet food was prepared in the same way control or high fat diet, but 0.1% bromophenol blue and 0.5% xylene cyanol was added to the recipe. Feeding assay experiments were performed as described in (Rajan and Perrimon, 2012). Groups of 5 adult males (in triplicate or quadruplicate) were placed on blue food, and control non-

blue food for background subtraction for 4 hours at 25°C, flies were then decapitated to avoid the potential effects the eye pigmentation may have on absorbance values. The resulting fly torsos were then homogenised in PBS and centrifuged for 20 minutes. Supernatant was collected, and absorbance measured at 625nm.

Confocal microscopy
A Leica SP5 microscope was used for imaging, using either the 10x, NA (numerical aperture) 0.4 objective, the 20x Dry NA 0.5 objective, or the 40x Oil NA 1.25 objective. Images were acquired with a resolution of either 1024x1024 or 512x512, at a scan speed of 400Hz. Between 3 and 6 line averages were carried out and tile scanning was used in order to image whole flies. Flies were anaesthetised during imaging using a built in CO_2 chamber. FACS sorted, fixed and stained plasmatocytes were imaged through glass coverslips using the 40x Oil NA 1.25 objective. Images were processed using Fiji and Imaris 7.3.0. Cell counts and co-localisation counts (DiI-LDL$^+$ and *HmlΔ*$^+$ plasmatocytes) were performed using the MATLAB spot detection function in Imaris 7.3.0. Sum fluorescence intensity (SFI) values were obtained using Fiji, for Oil Red O stained cells SFI was normalised to cell size. In *upd3*-GFP flies SFI was measured in 3 size matched areas per fly.

Injection calibration (For DiI-LDL injection and infection assays)
Microinjection needles for fly injection were produced in the lab using glass capillaries and a needle puller. Injection was performed using a PicospritzerR III, and injection volume was calibrated by injecting a drop into a plot of oil. Expelled drops were measured to obtain a final injection volume of 50 nanolitres (nl). Flies were anesthetised with CO_2 under a light microscope on porous gas pads prior to abdominal injection.

Bacterial infection
Stocks of *Micrococcus luteus* (*M. luteus*) and *Escherichia coli* (*E. coli*) were stored at -80°C, cultures were grown from these stocks by selecting a small quantity of bacteria using a pipette tip and dipping the tip in LB broth. Individual *E. coli* and *M. luteus* cultures were grown in a 37°C shaking incubator overnight. The following morning cultures were centrifuged at 4°C at 1600rpm for 10 minutes. Bacterial pellets were re-suspended in PBS and the optical density (OD) was measured at the wavelength of 600nm (OD600). Mixed septic infections were carried out with an equal mix of OD-adjusted *E. coli* and *M. luteus* cultures, retaining a final OD of 1. Injection, and drop calibration was performed as described above on adult male flies aged between 7 and 10 days. Experiments also included PBS injected flies, as wounding controls and non-injected controls. After infection or wounding, flies were homogenised in TRIzol and stored at -20°C ready for cDNA synthesis and RT qPCR, in this case flies were homogenised 6 hours post infection in order to observe maximal AMP induction.

DiI-LDL injection
Adult male reporter flies aged between 7 and 10 days were injected, as described above, with 50nl of DiI-LDL (3,3'-dioctadecylindocarbocyanine-low density lipoprotein from human plasma, Sigma-Aldrich). Flies were imaged with a confocal microscope 1 hour (*HmlΔ*) or 24 hours (*upd3*-GFP) post injection for plasmatocyte uptake of the DiI-LDL.

Woodcock et al., Revised *SUPPLEMENTAL* Figure 1.

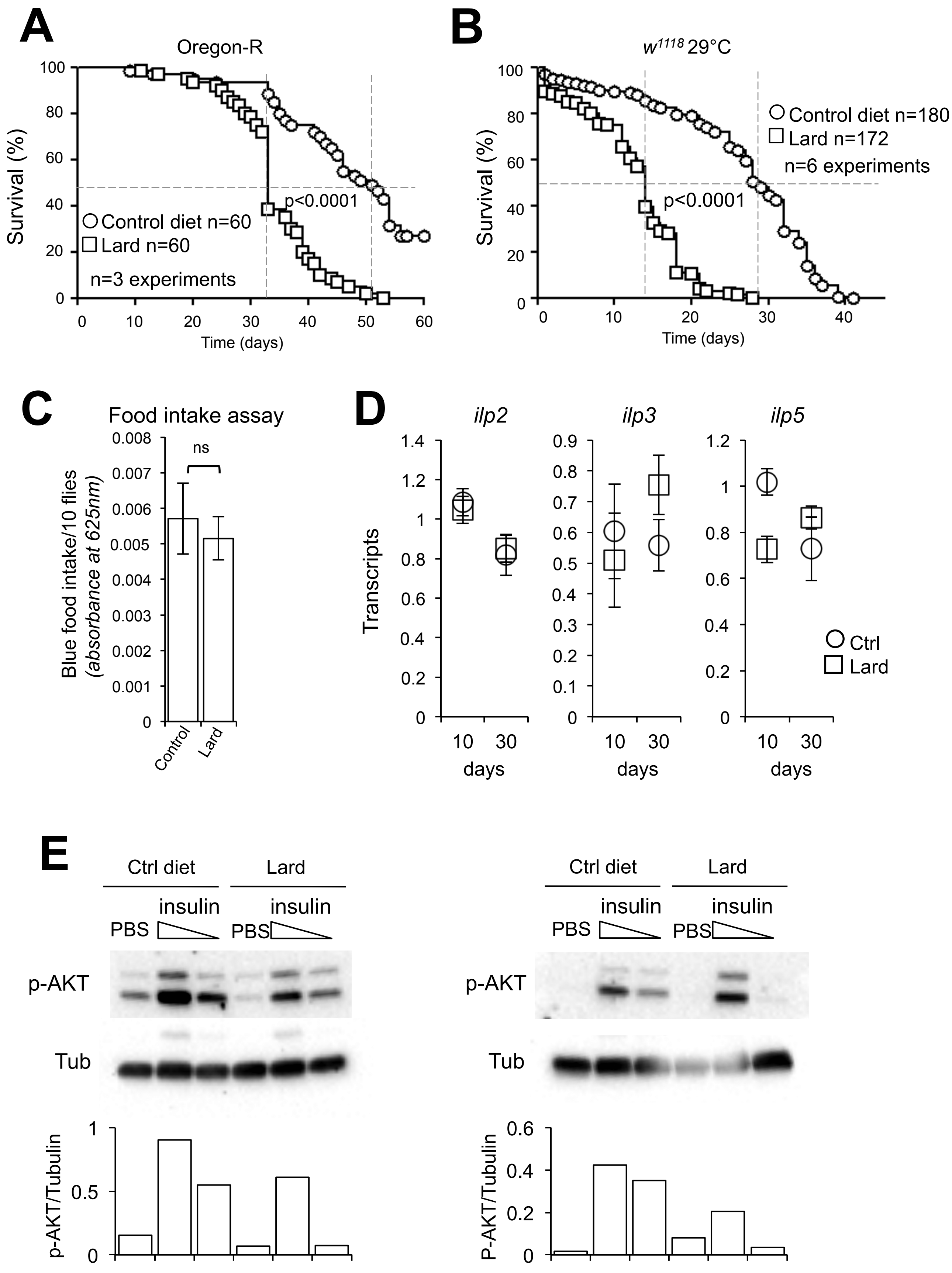

Woodcock et al., Revised *SUPPLEMENTAL* Figure 2.

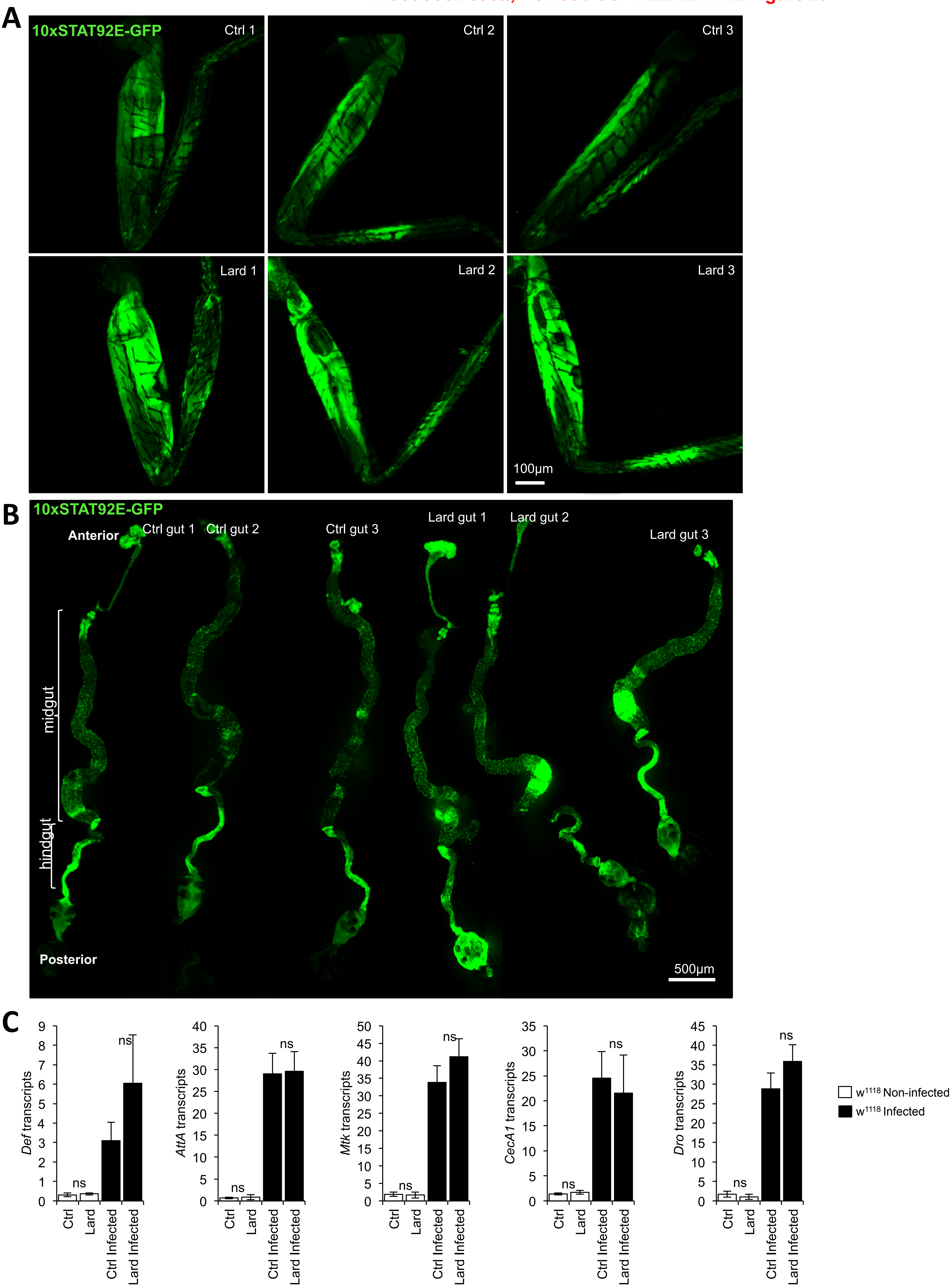

Woodcock et al., Revised *SUPPLEMENTAL* Figure 3.

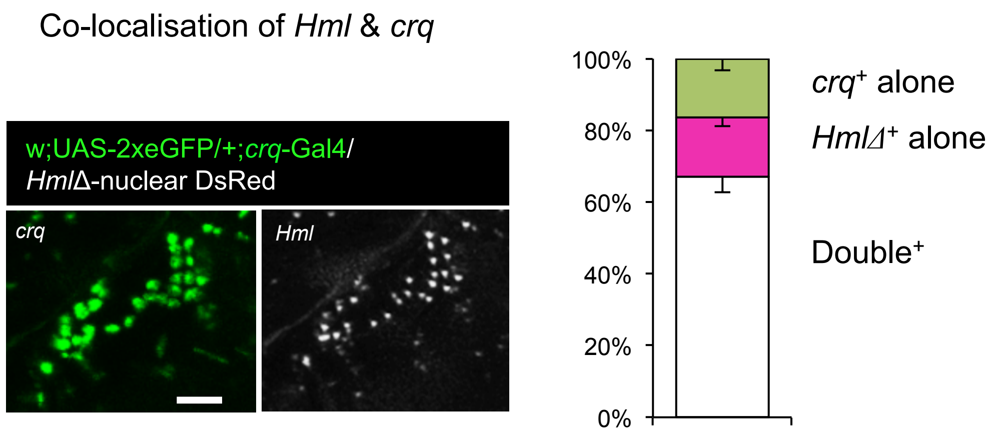

Woodcock et al., Revised *SUPPLEMENTAL* Figure 4.

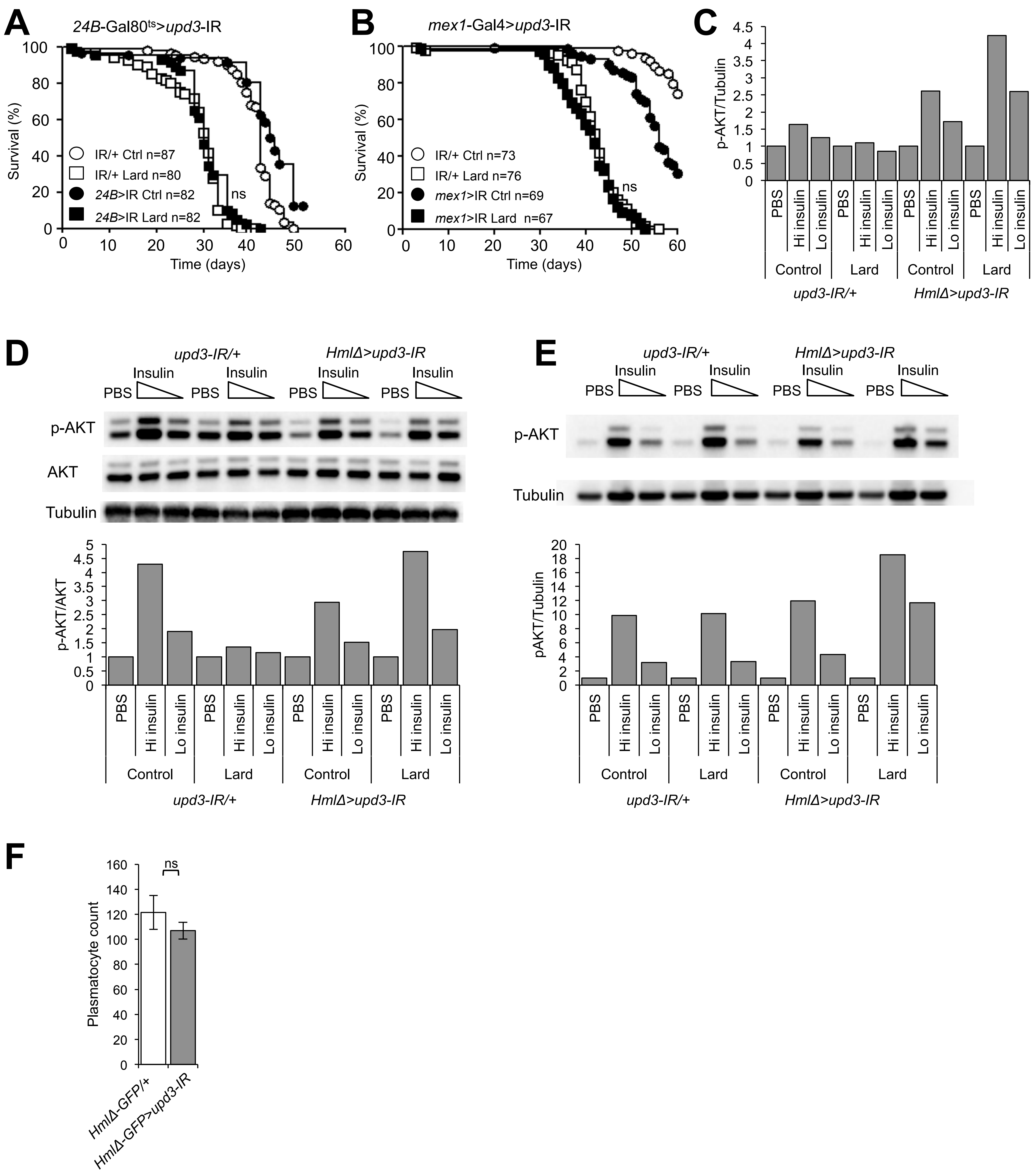

Woodcock et al., Revised *SUPPLEMENTAL* Figure 5.

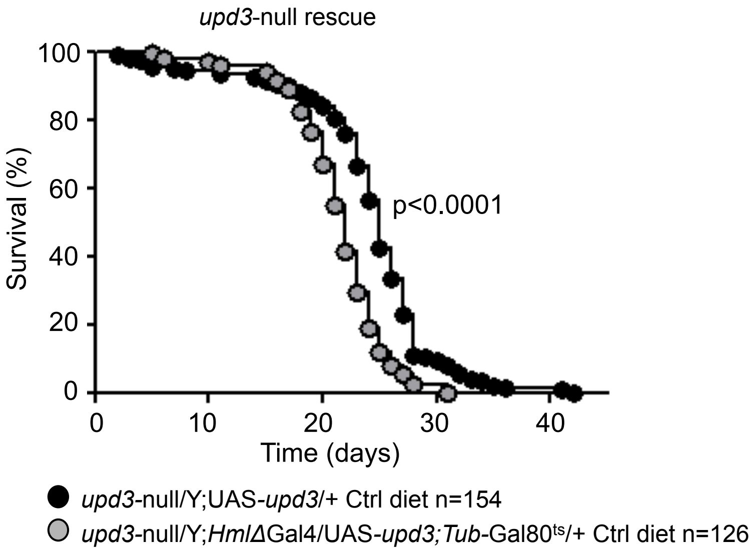

Log-rank X^2=44.40, P value P<0.0001
Wilcoxon X^2=39.36, P value P<0.0001

Woodcock et al., Revised *SUPPLEMENTAL* Figure 6.

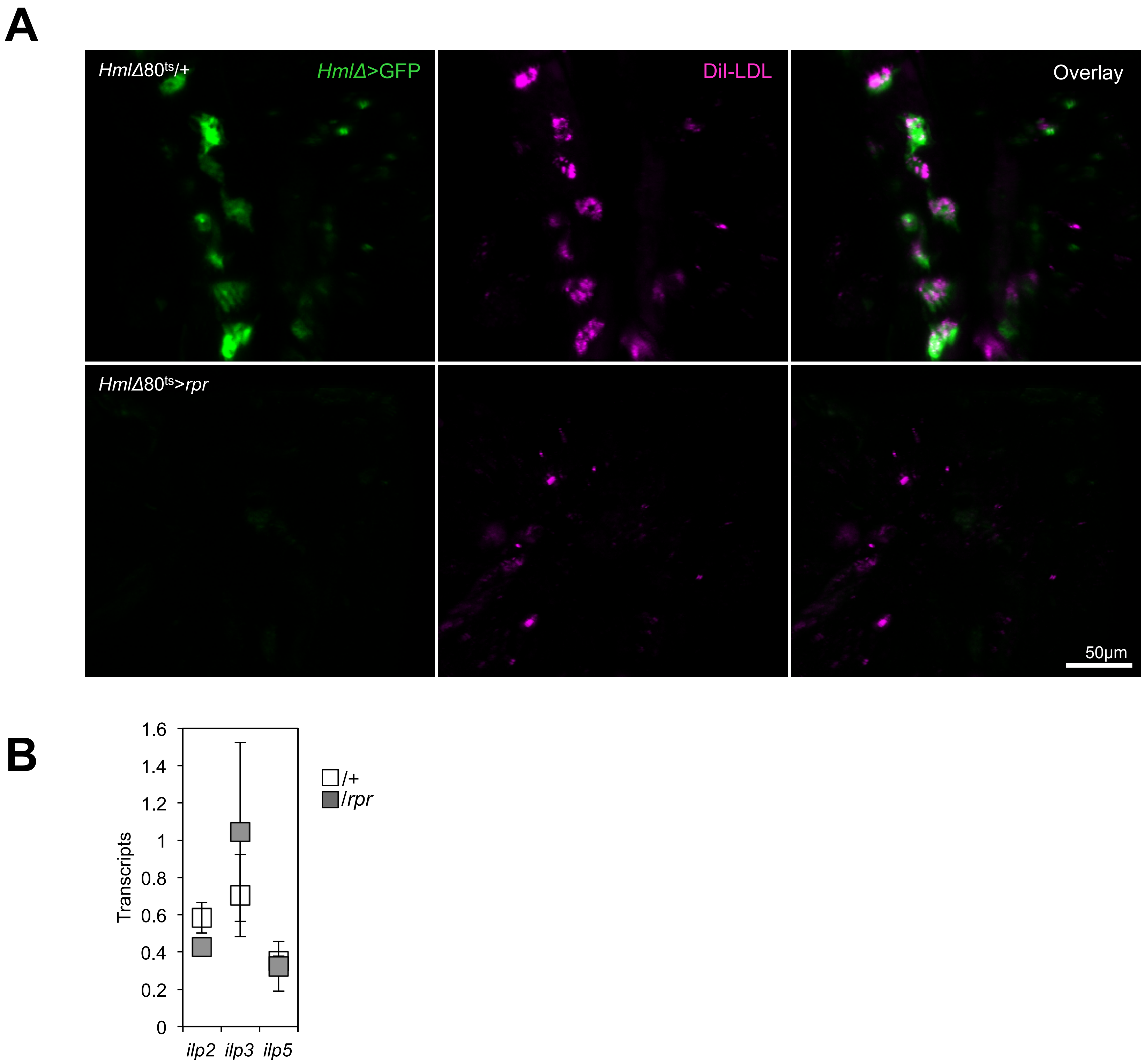

Woodcock et al., Revised *SUPPLEMENTAL* Figure 7.

A

Candidate gene	Expression in sorted hemocytes (qPCR)	Efficient knock down *HmlΔ*-Gal4	Efficient knock down with *Tub*-Gal4
Hemese	+	+	+
CG1887	No	Inconclusive	Not viable
CG2736	+	No	Not viable
NimC1	+	+	+
crq	+	+	+
CG3829	+	No	Not viable
CG7227	No	Inconclusive	Not viable
CG3212	No	No	No
peste	No	No	Not viable
CG10345	No	No	+
CG7422	No	Inconclusive	Not viable

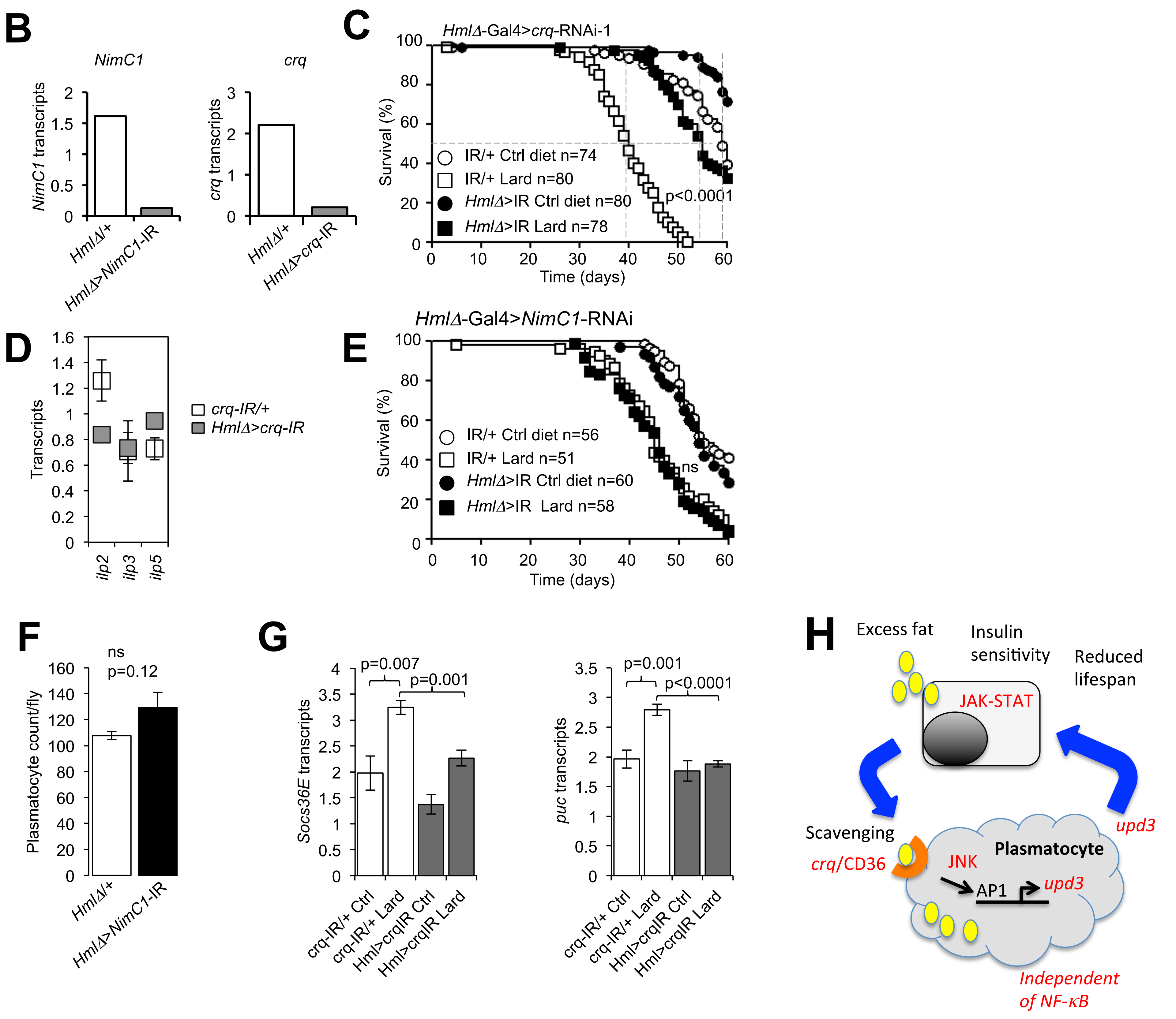

Supplement: Document S1. Supplemental Experimental Procedures and Figures S1–S7 [file mmc1.pdf]
