# Supplementary material for: Macrophage-Derived upd3 Cytokine Causes Impaired Glucose Homeostasis and Reduced Lifespan in Drosophila Fed a Lipid-Rich Diet
Source: Immunity. 2015 Jan 20;42(1):133–44. doi: 10.1016/j.immuni.2014.12.023 (PMC4304720; doi:10.1016/j.immuni.2014.12.023)

Immunity

Article

Macrophage-Derived *upd3* Cytokine Causes Impaired Glucose Homeostasis and Reduced Lifespan in *Drosophila* Fed a Lipid-Rich Diet

Graphical Abstract

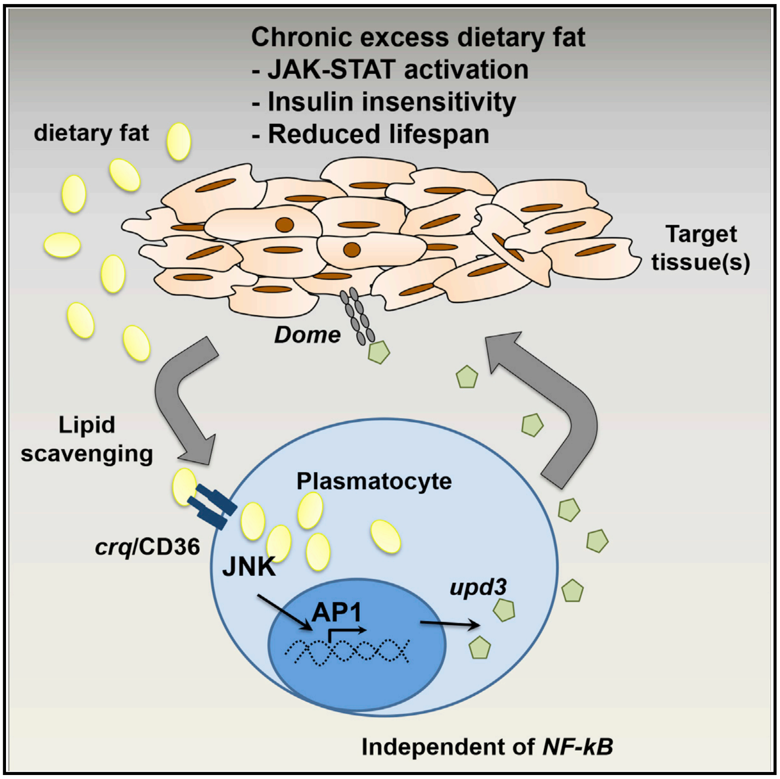

Authors

Katie J. Woodcock, Katrin Kierdorf, ..., Marc S. Dionne, Frédéric Geissmann

Correspondence

frederic.geissmann@kcl.ac.uk

In Brief

Consumption of fatty foods is associated across species with inflammation, metabolic imbalance, and reduced lifespan. Woodcock et al. use *Drosophila* fed a lipid-rich diet to demonstrate that an evolutionarily conserved “scavenger receptor-JNK-type 1 cytokine” cassette in macrophages controls glucose metabolism and reduces lifespan via activation of the JAK-STAT pathway.

Highlights

- Chronic lipid-rich diet results in JAK-STAT activation in *Drosophila*
- Chronic JAK-STAT activation reduces lifespan and insulin sensitivity
- Lipid-rich diet induces JNK pathway-dependent production of *upd3* by macrophages
- Macrophage *upd3* controls JAK-STAT activation, survival, and insulin sensitivity

Accession Numbers

GSE63254

Woodcock et al., 2015, Immunity *42*, 133–144
January 20, 2015 ©2015 The Authors
http://dx.doi.org/10.1016/j.immuni.2014.12.023

CellPress

Macrophage-Derived *upd3* Cytokine Causes Impaired Glucose Homeostasis and Reduced Lifespan in *Drosophila* Fed a Lipid-Rich Diet

Katie J. Woodcock,[1] Katrin Kierdorf,[1] Clara A. Pouchelon,[1] Valérie Vivancos,[1] Marc S. Dionne,[1,2] and Frédéric Geissmann[1,2,*]
[1]Centre for Molecular and Cellular Biology of Inflammation (CMCBI), Division of Immunity, Infection, and Inflammatory diseases, King's College London, London SE1 1UL, UK
[2]Co-senior author
*Correspondence: frederic.geissmann@kcl.ac.uk
http://dx.doi.org/10.1016/j.immuni.2014.12.023
This is an open access article under the CC BY-NC-ND license (http://creativecommons.org/licenses/by-nc-nd/4.0/).

SUMMARY

Long-term consumption of fatty foods is associated with obesity, macrophage activation and inflammation, metabolic imbalance, and a reduced lifespan. We took advantage of *Drosophila* genetics to investigate the role of macrophages and the pathway(s) that govern their response to dietary stress. Flies fed a lipid-rich diet presented with increased fat storage, systemic activation of JAK-STAT signaling, reduced insulin sensitivity, hyperglycemia, and a shorter lifespan. *Drosophila* macrophages produced the JAK-STAT-activating cytokine *upd3*, in a scavenger-receptor (*crq*) and JNK-dependent manner. Genetic depletion of macrophages or macrophage-specific silencing of *upd3* decreased JAK-STAT activation and rescued insulin sensitivity and the lifespan of *Drosophila*, but did not decrease fat storage. NF-κB signaling made no contribution to the phenotype observed. These results identify an evolutionarily conserved "scavenger receptor-JNK-type 1 cytokine" cassette in macrophages, which controls glucose metabolism and reduces lifespan in *Drosophila* maintained on a lipid-rich diet via activation of the JAK-STAT pathway.

INTRODUCTION

A lipid-rich diet has been associated with obesity and a reduced lifespan from *Drosophila* to humans (Birse et al., 2010; Driver and Cosopodiotis, 1979; Reilly and Kelly, 2011; Szendroedi and Roden, 2009; van Herpen and Schrauwen-Hinderling, 2008; Wagener et al., 2013). This represents a worldwide concern as societal changes have led to an increase in dietary lipid intake. Lipid-rich diets and obesity are associated with worldwide "epidemics" of cardiovascular diseases, type 2 diabetes, cancer, and inflammatory diseases in humans (Alikhani et al., 2013; Biswas and Mantovani, 2012; Kroenke et al., 2013; Moore and Tabas, 2011). Diseases associated with lipid-rich diets share common general features that include activation of the innate immune system, and in particular of macrophages, and the disruption of homeostasis (Biswas and Mantovani, 2012; Jin and Flavell, 2013; Moore and Tabas, 2011; Szendroedi and Roden, 2009; van Herpen and Schrauwen-Hinderling, 2008). Macrophage activation via innate pattern recognition receptors such as Toll-like receptor 4 (TLR4) and nuclear factor "kappa-light-chain-enhancer" of activated B cells (NF-κB) and the production of cytokines such as tumor necrosis factor-alpha (TNF-α), interleukin-1β (IL-1β), and interleukin-6 (IL-6) is proposed to mediate insulin resistance and other complications of obesity (Arkan et al., 2005; Biswas and Mantovani, 2012; Jin and Flavell, 2013; Moore and Tabas, 2011; Olefsky, 2009; Reilly et al., 2013). Nevertheless, the effects of the genetic deletion of Toll-like receptors such as TLR4 on insulin resistance are controversial (Jin and Flavell, 2013; Orr et al., 2012; Saberi et al., 2009; Tanti et al., 2012), and fat uptake by murine macrophages in vivo was also associated with suppression, rather than activation, of NF-κB-dependent inflammatory gene expression (Spann et al., 2012). The *cJun* NH2-terminal kinase (JNK), in macrophages, was also recently shown to be important for the establishment of diet-induced inflammation and insulin resistance in mice (Han et al., 2013; Hirosumi et al., 2002).

Causation studies in humans are challenging to conceive and to perform. Comprehensive genetic studies in mice are also arduous, in part due to their long duration, given rodent lifespans (Robertson et al., 2011), and difficulties in the interpretation of conventional knockout and cell depletion experiments (Clementi et al., 2009; Feng et al., 2011; Jin and Flavell, 2013; Orr et al., 2012; Saberi et al., 2009; Tanti et al., 2012). Therefore, the causative role of macrophages and the basic molecular mechanisms that may underlie their contribution to the disruption of homeostasis and lifespan reduction remain difficult to elucidate.

In contrast, *Drosophila melanogaster* is well suited to genetic analyses and survival studies (Dionne et al., 2006; Driver and Cosopodiotis, 1979; Heinrichsen and Haddad, 2012) and is an excellent model to explore homeostatic responses to stress, and in particular metabolic responses to diet (Birse et al., 2010). *Drosophila* has also proven to be a powerful model to decipher conserved mechanisms of innate immunity in metazoans (Lemaitre et al., 1996), and the adult fruit fly has a simple and genetically tractable myeloid immune system consisting of phagocytic macrophages, termed plasmatocytes (Agaisse et al., 2003;

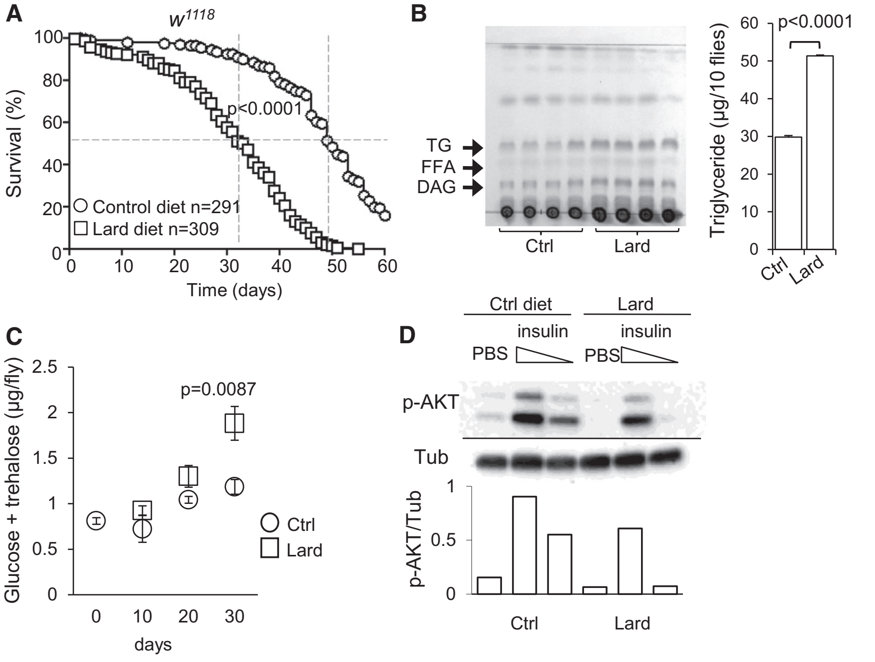

Figure 1. Reduced Lifespan and Systemic JAK-STAT Activation in *Drosophila* on Lipid-Rich Diet

(A) Survival of adult male flies (w^{1118}) fed a control diet (circles) or lard diet (squares). Log-rank test, $\chi^2 = 340.0$, $p < 0.0001$; Wilcoxon test, $\chi^2 = 271.9$, $p < 0.0001$. Data are pooled from six independent experiments. Dotted lines indicate 50% survival.

(B) Thin-layer chromatography (TLC) analysis of group of ten flies fed a control or lard diet for 10 days. TG, triglycerides; FFA, free fatty acids; DAG, diacyl glycerol. Histogram represents quantification of TG; n = 4; mean ± SEM.

(C) Glucose and trehalose content of flies fed control (circles) or lard diet (squares). Histograms represent mean ± SEM from four or five samples of three flies per diet.

(D) Amount of phospho-AKT 10 min after injection of PBS, 320 pg/fly (high) insulin, and 64 pg/fly (low) insulin in samples of three flies fed a control or lard diet for 30 days. Histogram represents quantification p-AKT over *Tubulin*, a representative experiment of three is shown. Please also see Figure S1.

Charroux and Royet, 2009; Gold and Brückner, 2014; Holz et al., 2003; Krzemień et al., 2010; Lebestky et al., 2000; Meister, 2004), allowing for genetic analysis of macrophage functions. We thus reasoned that *Drosophila* may be a favorable model to dissect evolutionarily conserved immune pathway(s) that may be at play in the macrophage response to excess dietary lipids and metabolic stress in vivo and to identify the roles of macrophages in longevity, glucose homeostasis, and fat storage.

We found that shorter lifespan, increased fat storage, reduced insulin sensitivity, and hyperglycemia were associated with systemic activation of the Janus kinase and signal transducer and activator of transcription (JAK-STAT) pathway, but not of the NF-κB pathway, in *Drosophila* maintained under a lipid-rich diet, while *Drosophila* macrophages became foamy and produced the type 1 cytokine *unpaired3* (*upd3*), which activates the JAK-STAT pathway. Using a combination of genetic approaches, we found that the production of *upd3* by macrophages required the scavenger receptor *croquemort* (*crq*) and JNK and was responsible for the systemic JAK-STAT activation, decreased insulin sensitivity, and the reduced lifespan of flies maintained on a lipid-rich diet. Fat storage itself was independent of macrophage activation and did not influence survival. These data demonstrate a causal role of macrophages in reducing insulin sensitivity and lifespan in flies maintained on a lipid-rich diet, via a pathway conserved in vertebrates.

RESULTS

Reduced Lifespan and Systemic JAK-STAT Activation in *Drosophila* on Lipid-Rich Diet

Extending earlier studies on the effect of dietary fat on metabolism and longevity in *Drosophila melanogaster* (Birse et al., 2010; Driver and Cosopodiotis, 1979; Heinrichsen and Haddad, 2012), we found that wild-type w^{1118} and Oregon-R *Drosophila* fed diets supplemented with lard and maintained at either 25°C or 29°C start dying 15–20 days earlier than flies on a control diet and thus show a decrease in lifespan of ~30% (Figure 1A; Figures S1A and S1B). Thin-layer chromatography (TLC) (Al-Anzi and Zinn, 2010) indicated that the total triglyceride content of lipid-rich diet-fed flies was increased compared to controls (Figure 1B), whereas food consumption was comparable (Figure S1C). *Drosophila* also presented with a progressive increase in glucose/trehalose, which doubled after 30 days of lipid-rich diet exposure (Figure 1C). *Ilp2*, *Ilp3*, and *Ilp5* (encoding insulin-like peptides) transcripts were unchanged (Figure S1D), but we observed a blunted phosphorylation of AKT in response to insulin, indicating that the lipid-rich diet resulted in impaired insulin sensitivity (Figure 1D; Figure S1E). Lipid-rich diet was also associated with an early and sustained increase of the cytokine *upd3* detectable at the whole-fly level (Figure 2A) and increased expression of the endogenous JAK-STAT target gene *Socs36E* (Figure 2B). Systemic JAK-STAT activation was confirmed by the analysis of 10XSTAT92E-GFP flies (Bach et al., 2007) where increased GFP expression was detected in muscle (Figure 2C) and midgut (Figure 2D; Figures S2A and S2B). In contrast, we did not detect activation of the transcriptional targets of the *Toll* and *Imd* NF-κB pathways in whole flies, although both the *Toll* and *Imd* pathways were responsive to sterile and septic injury (Figures 2E and 2F; Figure S2C). These data indicated that reduced lifespan and impaired glucose metabolism are associated with a systemic JAK-STAT activation in response to a lipid-rich diet in *Drosophila*.

Lipid-Rich Diet Promotes Upd3 Production by Macrophages in *Drosophila*

Tissue macrophages are professional phagocytes across the animal kingdom. *Drosophila* macrophages, termed plasmatocytes, are found throughout tissues of the adult fly (Meister,

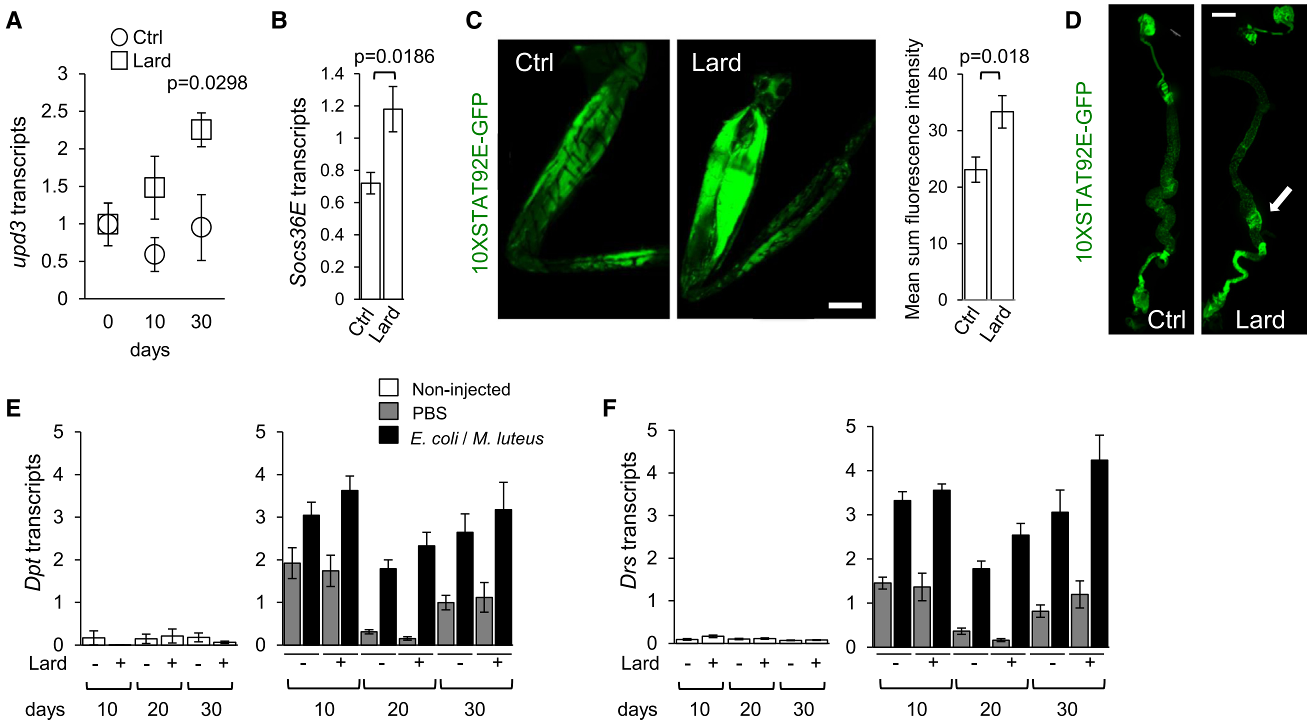

Figure 2. Systemic Activation of the JAK-STAT Pathway in Chronic Lipid-Rich Diet

(A) *upd3* transcription in flies fed a control or lard diet, three flies per sample, histogram represents mean ± SEM of four or five independent samples per diet and per time point.

(B) As in (A), *Socs36E* transcription in flies fed for 20 days.

(C) Confocal imaging analysis of GFP expression in legs from 10xSTAT92E-GFP flies fed control or lard diets for 10 days. Bar is 100 μm; a representative experiment is shown out of six. Histogram represents quantification of the mean sum fluorescence intensity ± SEM for six flies per diet.

(D) As in (C), STAT92E-GFP expression in guts. Arrow represents area of enhanced STAT92E-GFP expression in the midgut. Bar is 300 μm. Representative experiment is shown out of eight flies per diet. See also Figure S2.

(E and F) *Drs* and *Dpt* transcripts in unchallenged (white bars), PBS-injected (gray bars), and *E. coli*-and *M. luteus*-infected flies (black bars) after 10, 20, and 30 days of control or lard-enriched diets. Histograms represent mean ± SEM of 4–5 independent samples per diet, per time point. Please also see Figure S2.

2004; Figure 3A) and are left over from embryonic and larval hematopoiesis (Holz et al., 2003; Krzemień et al., 2007, 2010). They express lectins and scavenger receptors such as *Hemolectin* (*Hml*) (Goto et al., 2001) and *crq*, a fly *CD36* homolog (Franc et al., 1999; Stuart et al., 2005; Figure S3). *Drosophila* macrophages changed morphology on lipid-rich diet (Figure 3B). Their numbers were not modified as a result of the diet (Figure 3C), but analysis of fluorescence-activated cell sorting (FACS)-sorted plasmatocytes indicated they become foamy, accumulating neutral triglycerides and lipids within large Oil red O-positive lipid vacuoles (Figure 3D), a feature also typical of the tissue macrophage response to a lipid-rich diet in vertebrates (Li and Glass, 2002). Whole-genome expression array analysis indicated that expression of a large number of genes was upregulated or downregulated in macrophages from flies on a lipid-rich diet after 30 days, in comparison to cells from control diet-fed flies (Figure 3E). Transcription-factor binding site representation analysis (Clark et al., 2013) identified an overrepresentation of AP-1, Fox, and ATF-CREB binding sites, but not NF-κB binding sites, in the vicinity of upregulated genes (Figure 3F). A qPCR analysis of FACS-sorted plasmatocytes did not detect activation of the transcriptional targets of the *Toll* and *Imd* NF-κB pathways, *Drosomycin* (*Drs*) and *Diptericin*, but indicated that macrophages from flies on a lipid-rich diet produced the JAK-STAT activating cytokine *upd3*, but not *upd* and *upd2* (Figure 3G). *Upd*, *upd2*, and *upd3* share a common gp130-like receptor, *domeless* (*dome*), that signals via a single JAK (*hop*) and a single STAT (*Stat92E*) (Agaisse and Perrimon, 2004; Binari and Perrimon, 1994; Brown et al., 2001; Hou et al., 1996; Yan et al., 1996). These data indicated that lipid-rich diet promotes *Drosophila* macrophage activation characterized by the production of *upd3*.

Macrophage-Derived Upd3 Is Responsible for the Metabolic and Lifespan Phenotype on Lipid-Rich Diet

Upd3 was previously shown to be produced by macrophages in response to infection (Agaisse et al., 2003), but *upd3* is also produced by several other cell types and tissues in *Drosophila*, including midgut epithelial cells, where it plays an essential role in gut infection and homeostasis (Buchon et al., 2009; Jiang et al., 2009; Osman et al., 2012). To investigate whether *upd3* produced by macrophages or from other sources might be responsible for the phenotype of flies under a lipid-rich diet, we generated tissue-specific *upd3* knockdown flies. Silencing of *upd3* in the midgut or muscle did not rescue fly longevity (Figures S4A and S4B). However, silencing of *upd3*

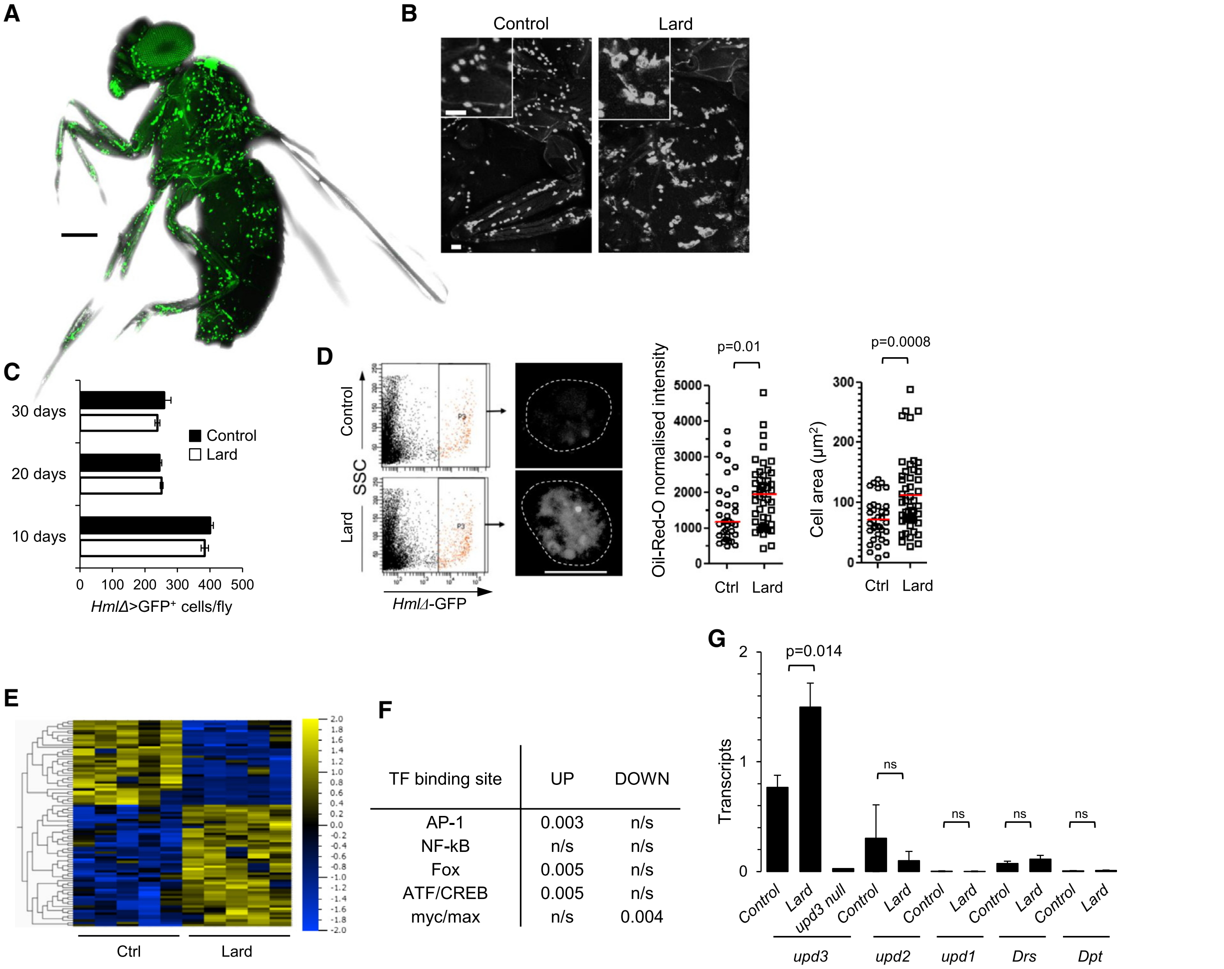

Figure 3. Macrophage Activation in Response to Chronic Lipid-Rich Diet

(A) Confocal microscopy image (tiled/z stack, see Supplemental Experimental Procedures) of GFP+ plasmatocytes in 2-week-old w^{1118};*HmlΔ*-Gal4,UAS-2xeGFP male. Representative of more than ten flies. Bar is 250 μm. See also Figure S3.

(B) As in (A), in flies maintained on control or lard diet for 10 days. Bars, 50 μm.

(C) Number of GFP+ plasmatocytes in flies (as in A) fed a control or lard diet for 10, 20, or 30 days. Histograms show mean ± SEM from eight flies per diet and time point.

(D) Analysis of oil red O lipid staining intensity and cell area of FACS-sorted plasmatocytes from flies fed a control (n = 35 cells) or lard diet (n = 49 cells) for 20 days. Images are analyzed by confocal microscopy; bar is 7.5 μm. Symbols represent values for individual cells; red bar indicates the mean. Plasmatocytes analyzed were collected from > 4 independent experiments.

(E and F) Whole-genome expression array (Agilent) analysis of plasmatocytes from flies fed a normal or lard diet for 30 days. (E) Heat map represents up- and downregulated annotated genes across five independent biological replicates (fold change > 2, p < 0.01). (F) Transcription-factor binding sites identified as overrepresented in groups of genes shown in (E). "Fox" refers to a generic FOX (winged-helix) binding site.

(G) *upd3*, *upd2*, *upd*, *Drs*, *and Drs1* transcripts in FACS-sorted plasmatocytes from w^{1118};*HmlΔ*-Gal4,UAS-2xeGFP flies fed a control or lard diet for 20 days. Histogram represents mean ± SEM from four or five samples of 20,000 cells per diet and genotype. *Upd3* null represents *upd3* transcription in *upd3* null sorted plasmatocytes under control diet. Please also see Figure S3.

in macrophages improved fly longevity (Figure 4A) and decreased *upd3* induction in response to lipid-rich diet at whole-fly level (Figure 4B). Furthermore, macrophage-specific *upd3* knockdown also decreased glucose/trehalose almost to control concentrations (Figure 4C), and restored AKT phosphorylation in response to insulin (Figure 4D; Figures S4C–S4E). *Ilp2*, *Ilp3*, and *Ilp5* transcription was stable (Figure 4E), and plasmatocyte numbers were unchanged (Figure S4F). In addition, macrophage-specific *upd3* knockdown did not affect fly fat content (Figure 4F).

These data suggested that *Drosophila* macrophages were responsible for reduced lifespan and the disruption of glucose homeostasis, via their production of *upd3*. To confirm whether the production of *upd3* was indeed responsible for systemic JAK-STAT activation and for the survival and metabolic phenotypes, we examined *Drosophila* genetically deficient for *upd3* (*upd3* null) (Osman et al., 2012). The lifespan of *upd3* null *Drosophila* was identical on control and lipid-rich diets (Figure 5A). *Upd3* null flies did not present with increased JAK-STAT activation in the course of a lipid-rich diet (Figure 5B)

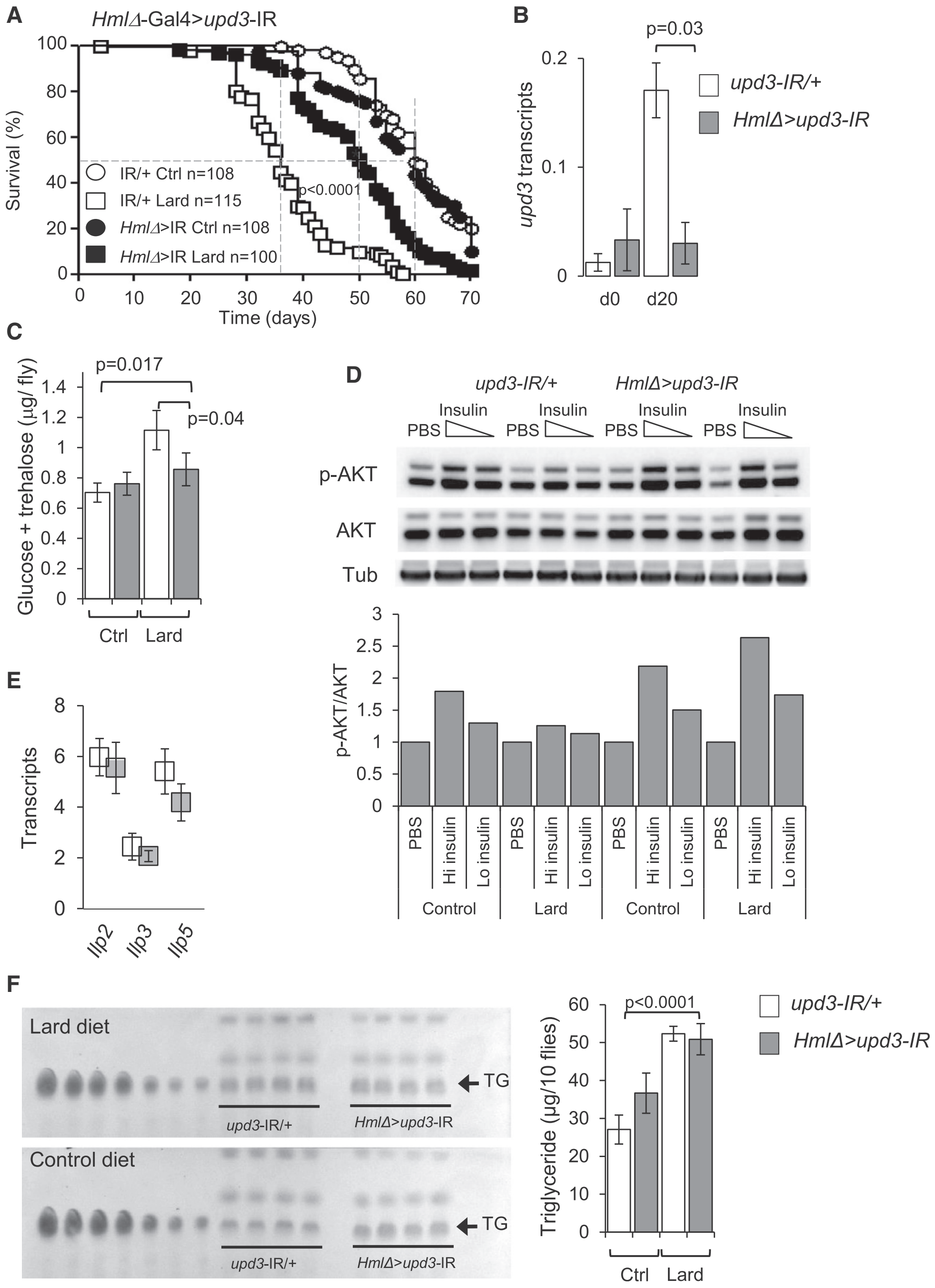

Figure 4. Macrophage-Derived Upd3 Controls Survival and Insulin Sensitivity in Lipid-Rich Diet

(A) Survival of plasmatocyte (*HmlΔ*)-driven *upd3* knockdown flies (*HmlΔ*-Gal4,UAS-2xeGFP/UAS-*upd3*-IR, closed symbols) and control flies (UAS-*upd3*-IR/+, open symbols) on control or lard diet. Data are pooled from five independent experiments. Log-rank, $\chi^2 = 68.79$, $p < 0.0001$; Wilcoxon, $\chi^2 = 65.83$, $p < 0.0001$ for flies under lard diet.

(B) *Upd3* transcription in flies as in (A) at days 0 and 20. Histogram shows mean ± SEM of four or five independent samples of three flies per diet and genotype.

(C) Total glucose and trehalose content of flies as in (A) on a control or lard diet for 40 days. Histogram represents mean ± SEM from four or five samples of three flies per diet.

(D) Amount of phospho-AKT in flies as in (A) 10 min after injection of PBS, 320 pg/fly (high) insulin, and 64 pg/fly (low) insulin in samples of three flies fed a control or lard diet for 40 days. Histogram represents quantification p-AKT over total AKT, n = 3; see also Figure S4.

(E) *Ilp2*, *Ilp3*, and *Ilp5* transcription in *upd3*-IR and control flies fed a lard diet. Three flies per sample; histogram represents mean ± SEM of four or five independent samples per diet and per time point.

(F) TLC for TG contents of groups of ten flies as in (A) fed a control or lard diet for 20 days. Histogram represents quantification of TG; n = 4, mean ± SEM. Please also see Figure S4.

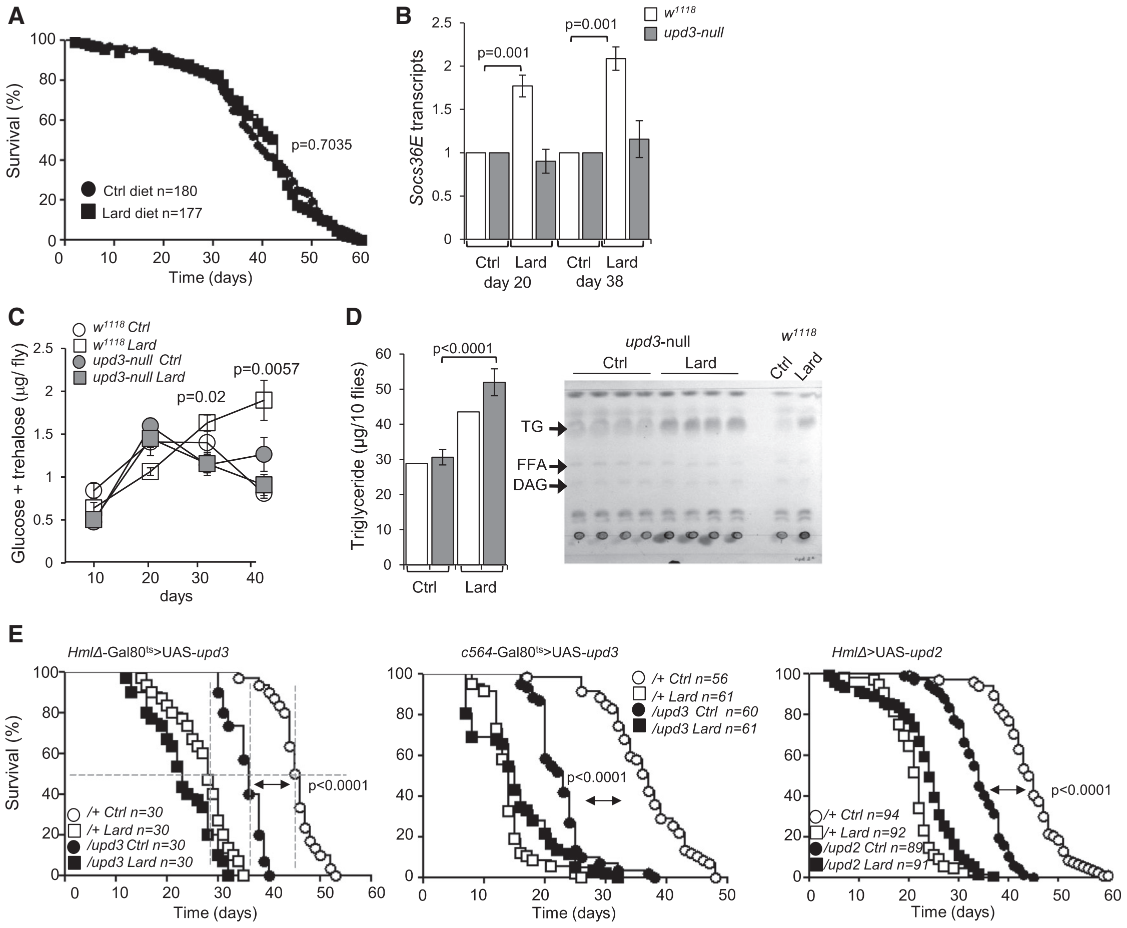

Figure 5. Chronic Lipid-Rich Diet Does Not Affect Lifespan and Glucose Level in Upd3 Null *Drosophila*

(A) Survival of *upd3* null on control and lard diets. Log-rank, $\chi^2 = 0.2264$, $p = 0.6342$; Wilcoxon, $\chi^2 = 0.1449$, $p = 0.7035$. Data are pooled from eight independent experiments.

(B) *Socs36E* transcripts in *upd3* null flies and w^{1118} controls. Three flies per sample; histogram represents mean ± SEM of four or five independent samples per diet and per time point.

(C) Time course of glucose and trehalose concentrations in flies as in (B) on a control or lard diet. Histograms represent mean ± SEM from four or five samples of three flies per diet.

(D) TLC analysis of TG content of *upd3* null flies and w^{1118} controls fed a control or lard diet for ten days. Histogram represents quantification of TG; n = 4, mean ± SEM.

(E) Left: Survival of inducible plasmatocyte (*HmlΔ*)-driven overexpression of *upd3* (*HmlΔ*Gal80^{ts} > upd3, closed shapes) compared to control flies (UAS-*upd3*/+, open shapes). Log-rank, $\chi^2 = 56.16$, $p < 0.0001$; Wilcoxon, $\chi^2 = 46.46$, $p < 0.0001$ for flies fed a control diet. Center: Survival of inducible fat body (c564)-driven overexpression of *upd3* (c564Gal80^{ts} > *upd3*, closed shapes) compared to controls (UAS-*upd3*/+, open shapes). Data are pooled from two independent experiments. Log-rank, $\chi^2 = 97.38$, $p < 0.0001$; Wilcoxon, $\chi^2 = 88.73$, $p < 0.0001$ for flies fed a control diet. Right: Survival of plasmatocyte (*HmlΔ*)-driven overexpression of *upd2* (*HmlΔ* > *upd2*, closed shapes) compared to controls (UAS-*upd2*/+, open shapes). Data are pooled from three independent experiments. Log-rank, $\chi^2 = 119.5$, $p < 0.0001$; Wilcoxon, $\chi^2 = 102.7$, $p < 0.0001$ for flies fed a control diet. Please also see Figure S5.

and did not develop hyperglycemia (Figure 5C), although they stored triglycerides when fed lipid-rich diets (Figure 5D). Plasmatocyte-specific expression of *upd3* in *upd3 null* flies reduced their lifespan on a control diet (Figure S5), and ectopic inducible expression of *upd3* or of *upd2*, in plasmatocytes or in the fat body, was also sufficient to reduce *Drosophila* lifespan (Figure 5E).

Altogether, these data suggested that, in *Drosophila* maintained on lipid-rich diet, macrophages are a major source of *upd3* production, which is responsible for systemic activation of the JAK-STAT pathway, reduced lifespan, and the disruption of glucose homeostasis.

Drosophila Macrophages Control Glucose Homeostasis and Survival of Flies on Lipid-Rich Diet

To further challenge this hypothesis, we generated inducible macrophage-depleted adult flies (Charroux and Royet, 2009). Cell death was triggered in adult flies by inducible expression of the proapoptotic protein *reaper* (*rpr*) (White et al., 1996) in macrophages under the control of *HmlΔ* combined with a temperature-specific repressor (McGuire et al., 2004). Injected fluorescent lipids (DiI-LDL) are rapidly scavenged by plasmatocytes, and there was a strong correlation between the DiI and *HmlΔ*$^+$ cells 1 hr after injection (Figure 6A). Furthermore, expression of *upd3*-GFP increased in plasmatocytes of flies

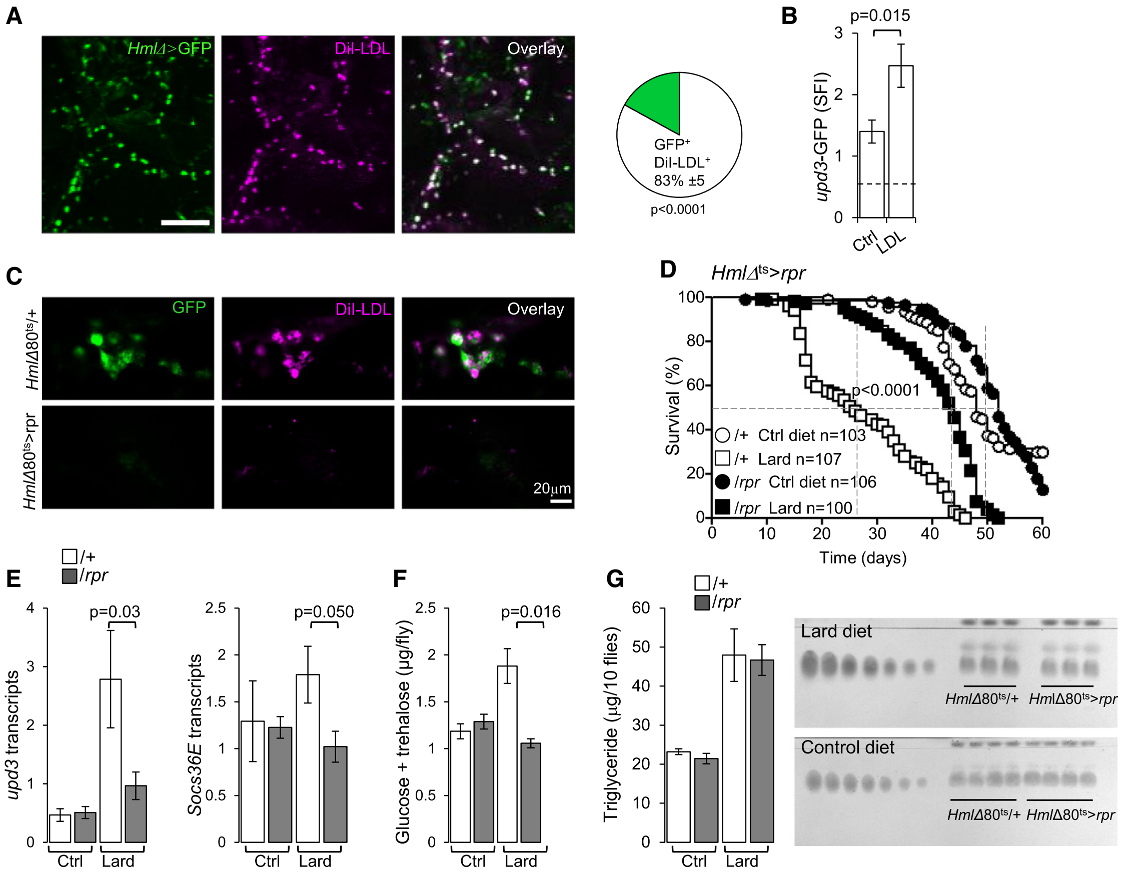

Figure 6. Macrophage Depletion in Lipid-Rich Diet Rescues *Drosophila* Lifespan and Glucose Metabolism

(A) DiI-LDL (magenta) uptake 1 hr after injection by *HmlΔ*⁺ plasmatocytes (green). White cells represent the overlay of GFP and DiI-LDL. Bar, 100 μm. Pie chart: percent colocalization of *HmlΔ*⁺ cells with DiI-LDL, mean ± SD, $p < 0.0001$, n = 3.

(B) Sum GFP fluorescence intensity in *upd3*-Gal4,UAS-GFP reporter flies injected with DiI-LDL or PBS. Mean ± SEM, n = 4.

(C) Confocal images of plasmatocyte-depleted and control flies injected with DiI-LDL (see Experimental Procedures). n = 4.

(D) Survival of plasmatocyte-depleted (*HmlΔ*Gal80^{ts} > *rpr*, closed shapes) and control flies (*HmlΔ*Gal80^{ts}/+, open shapes) fed a control or lard diet. Data are pooled from four independent experiments. Log-rank, $\chi^2 = 75.53$, $p < 0.0001$; Wilcoxon, $\chi^2 = 68.46$, $p < 0.0001$ for flies fed a lard diet.

(E) *upd3* and *Socs36E* transcripts in flies as in (D) fed lard or control diets for 20 days; three flies per sample; histogram represents mean ± SEM of four or five independent samples per diet.

(F) Total glucose and trehalose content of flies as in (D) on control or lard diet for 30 days. Histograms represent mean ± SEM from four or five samples of three flies per diet.

(G) TLC analysis of triglyceride contents of groups of ten flies as in (D) fed control or lard diets for 20 days. Histogram represents quantification of triglyceride (TG); n = 4, mean ± SEM. Please also see Figure S6.

injected with DiI-LDL (Figure 6B). Following *rpr* induction, macrophage depletion was complete as assessed by the absence of lipid scavenging in the flies (Figure 6C; Figure S6A). Macrophage depletion extended the longevity of flies on lipid-rich diet (Figure 6D), and decreased *upd3* and *Socs36E* expression (Figure 6E). Macrophage-depleted flies were also protected from hyperglycemia in comparison to controls (Figure 6F). Macrophage depletion did not affect the triglyceride content of the flies (Figure 6G), and *Ilp2*, *Ilp3*, and *Ilp5* transcription remained stable (Figure S6B). Our data thus indicated that macrophages are responsible for the reduced lifespan of *Drosophila* under lipid-rich diet, via the production of *upd3*. We therefore investigated the pathways and molecules that drive *upd3* production in *Drosophila* macrophages.

Upd3 Production by Macrophages Is Controlled by Croquemort and JNK, but Not by NF-κB Signaling

The above data suggested that lipid uptake by macrophages may regulate *upd3* production. We thus screened the fly genome for putative scavenger receptors involved in lipid uptake and expressed by macrophages (Figure S7A) and tested their roles in *upd3* production and survival. Plamatocyte-specific knockdown of two scavenger receptors, a fly *CD36* homolog *crq* (Franc et al., 1999; Stuart et al., 2005) and the EGF-like repeat containing scavenger receptor *Nimrod C1* (*NimC1*) (Kurucz et al., 2007) (Figures S7A and S7B), resulted in a 30%–40% reduction in macrophage lipid uptake (Figure 7A). Knockdown of *crq* decreased *upd3* and *Socs36E* expression in the whole fly (Figure 7B) and extended *Drosophila* lifespan (Figure 7C; Figure S7C), yet *Ilp2*, *Ilp3*, and *Ilp5* transcription was not significantly changed

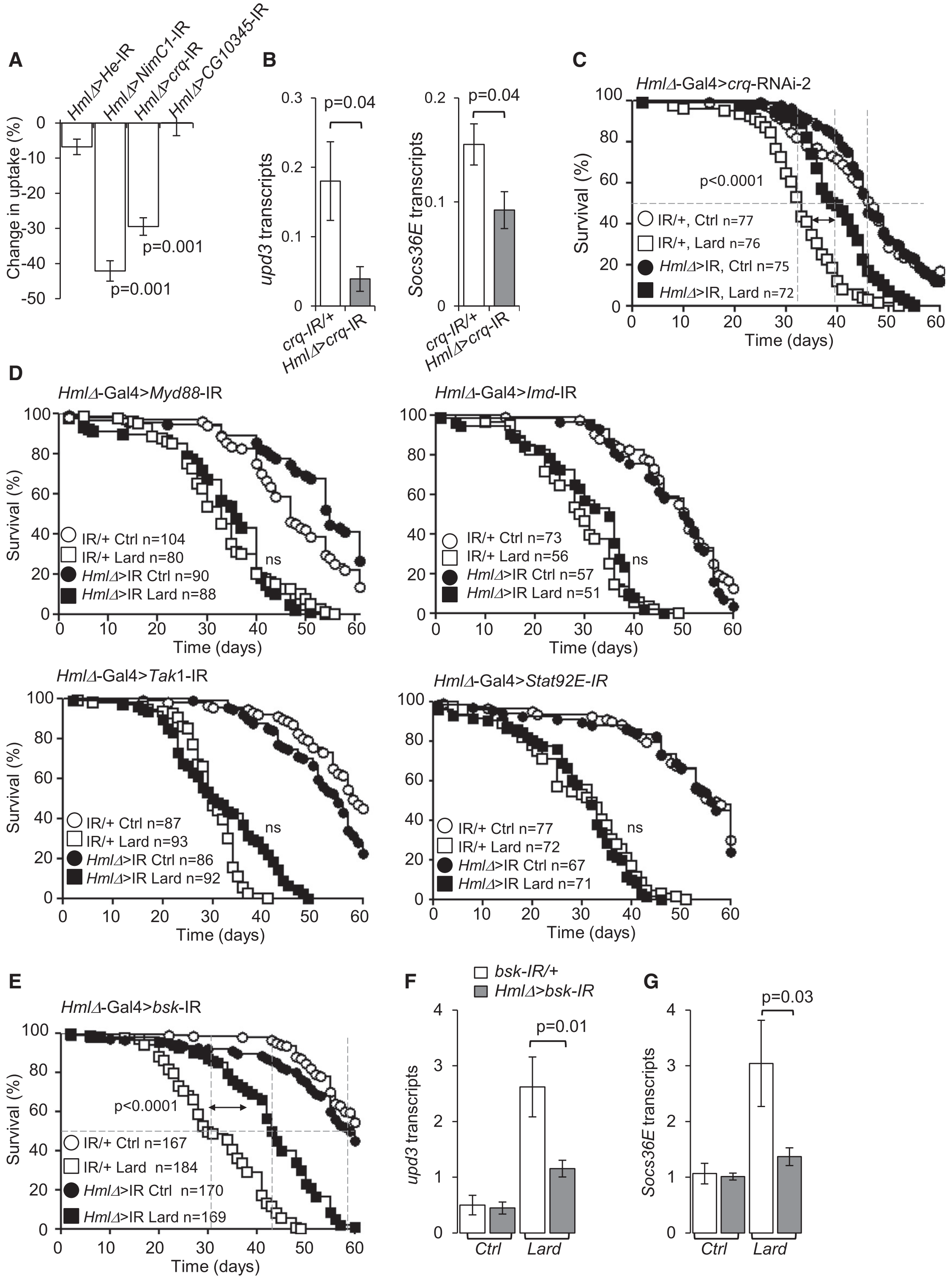

Figure 7. Control of Upd3 Expression by Macrophages during Lipid-Rich Diet

(A) Percentage reduction in DiI-LDL uptake as compared to controls in plasmatocyte-specific scavenger receptor knockdown flies (*crq* and *NimC1*) and in *Hemese* (*He*) and *CG10345* knockdown flies (see Figure S7A and Experimental Procedures). Histograms represent mean ± SEM; n = 4 flies per genotype.

(B) *upd3* and *Socs36E* transcripts in plasmatocyte (*HmlΔ* > *crq*-IR, gray) and control (UAS-*crq*-IR/+, white) flies fed lard diets for 20 days; three flies per sample; histogram represents mean ± SEM of four or five independent samples per diet.

(legend continued on next page)

(Figure S7D). However, knockdown of *NimC1* in macrophages did not influence the lifespan of flies on a lipid-rich diet (Figure S7E).

The lack of effect of *NimC1* knockdown on survival despite its effect on lipid uptake suggested that the effect of *crq* knockdown on survival may be linked to signaling. We thus investigated the roles of signaling pathways that may be involved in the expression of *upd3* by macrophages. Silencing of myeloid differentiation primary response gene 88 (*Myd88*), immune deficiency (*Imd*), and TGF-β-activated kinase 1 (*Tak1*) in macrophages (Figure 7D) did not rescue *Drosophila* lifespan on lipid-rich diet. These data were in accordance with the lack of detectable NF-κB activation in macrophages (see Figures 2E, 2F, 3F, and 3G). In contrast, we found that silencing of JNK (*bsk*) in macrophages (*HmlΔ*-Gal4 > *bsk*-IR) extended the longevity of flies maintained under a lipid-rich diet (Figure 7E) and decreased expression of *upd3* (Figure 7F) and *Socs36E* (Figure 7G) in the whole fly, but did not affect plasmatocyte numbers (Figure S7F). This result was consistent with the observed overrepresentation of AP-1 but not NF-κB binding sites in the vicinity of genes upregulated in plasmatocytes from lipid-rich diet-fed flies (Figure 3F). Additionally, silencing of signal-transducer and activator of transcription protein at 92E (*Stat92E*) in macrophages did not rescue *Drosophila* longevity on lipid-rich diet (Figure 7D), suggesting that JNK acts downstream of *crq*, but upstream of *upd3* (Figure S7H). Indeed, regulation of the JNK transcriptional target puckered (puc) on a lipid-rich diet was lost in *crq* knockdown flies (Figure S7G). The regulation of *upd3* expression by JNK in macrophages is reminiscent of the previously described regulation of *upd3* expression by JNK (Jiang et al., 2009) but not NF-κB signaling (Buchon et al., 2009) in enterocytes from the *Drosophila* midgut.

DISCUSSION

Collectively, our observations in a simple model organism demonstrate an essential role of macrophages in the loss of glucose homeostasis and lifespan reduction caused by a chronic lipid-rich diet, via the prolonged production of the type 1 cytokine *upd3* that activates the JAK-STAT pathway. Further, we identify a genetic *crq-JNK-upd3* cassette in macrophages critical for the longevity of "obese" flies. Our findings suggest that scavenging of excess lipids via *crq*, a CD36 analog (Stuart et al., 2005), results in the activation of a JNK-mediated stress response in macrophages, which in turn drives their production of *upd3*, activating the JAK-STAT pathway, which controls insulin sensitivity and lifespan in *Drosophila*.

It is interesting to note that this response is distinct from a NF-κB-dependent immune response, since reduced longevity and insulin resistance due to lipid-rich diet were not caused by transcription of NF-κB target genes in the whole fly or in macrophages. These data are consistent with recent evidence that lipid uptake by murine macrophages does not activate NF-κB (Spann et al., 2012). It is also noteworthy that the lipid storage phenotype was not affected by macrophage depletion or genetic reduction of *upd3* production and JAK-STAT signaling. This suggests that, in the fly, the macrophage stress response mediated by cytokine production causes disease, rather than lipid storage itself.

The JAK-STAT pathway and *upd3* in *Drosophila* are involved in the maintenance of tissue homeostasis and tissue repair in conditions of acute stress and infection via the control of stem cell proliferation in the *Drosophila* gut, testis, ovary, and kidney (Jiang et al., 2009; Kohlmaier and Edgar, 2008; Osman et al., 2012; Singh et al., 2007); the inhibition of apoptosis (Betz et al., 2008); and the regulation of *Ilp* secretion (Rajan and Perrimon, 2012) and insulin signaling (Karpac et al., 2011). Reducing insulin-TOR activity in *Drosophila* decreases cardiac dysfunction induced by short-term lipid-rich diet (Birse et al., 2010). Paradoxically, we found here that sustained production of *upd3* in response to chronic excess dietary lipid caused impaired glucose metabolism and decreased lifespan. Thus JAK-STAT activation may contribute to the regulation of energy storage, at least in part through insulin receptor signaling, a protective effect that may nevertheless affect host fitness and cause pathogenesis, depending on its magnitude and duration (Jamieson et al., 2013; Medzhitov et al., 2012).

Vertebrate CD36 is involved in lipid scavenging (Stuart et al., 2005), and activates JNK (Baranova et al., 2008; Rahaman et al., 2006), while mice with JNK-deficient macrophages present with decreased production of cytokines, including IL-6, and are protected against insulin resistance (Han et al., 2013). Therefore, we hypothesize that a conserved mechanism by which excess lipids activate a chronic stress response mediated by macrophage scavenger receptors, JNK, and type 1 cytokine production may cause dysregulated metabolic homeostasis and reduced lifespan and represents a target for therapeutic interventions.

EXPERIMENTAL PROCEDURES

Drosophila melanogaster Stocks

See Supplemental Experimental Procedures.

Lipid-Rich Diet Preparation

Lipid-rich diets were prepared using the control diet as a base recipe and adding lard (protein, nil; carbohydrates, nil; saturated fat, 44%; monounsaturated,

(C) Survival of plasmatocyte-specific *crq* knockdown flies compared to controls as in (B) on control or lard diet. Data pooled from four independent experiments. Log-rank, χ^2 = 31.15, p < 0.0001; Wilcoxon, χ^2 = 32.75, p < 0.0001 for flies on a lard diet.

(D) Survival of plasmatocyte (*HmlΔ*)-specific *Myd88* (top left), *Imd* (top right), *Tak1* (bottom left), and *Stat92E* (bottom right) knockdown flies (closed shapes) and controls (open shapes) on a control or lard diet. *HmlΔ* > *MyD88*-IR data were pooled from three independent experiments. Log-rank, χ^2 = 0.000000002953, p = 1.0000; Wilcoxon, χ^2 = 1.124, p = 0.2891 for flies on a lard diet. *HmlΔ* > *Imd*-IR: data were pooled from three independent experiments. Log-rank, χ^2 = 2.104, p = 0.1469; Wilcoxon χ^2 = 2.872, p = 0.0901 for flies on a lard diet. *HmlΔ* > *Tak1*-IR data were pooled from three independent experiments. Log-rank, χ^2 = 1.124, p = 0.2891; Wilcoxon, χ^2 = 1.138, p = 0.2862 for flies on a lard diet. *HmlΔ* > *Stat92E*-IR data are pooled from three independent experiments. Log-rank, χ^2 = 1.011, p = 0.3146; Wilcoxon, χ^2 = 0.03924, p = 0.8430 for flies fed a lard diet.

(E) Survival of plasmatocyte-specific *bsk* knockdown flies (*HmlΔ* > *bsk*-IR, closed shapes) and control flies (UAS-*bsk*-IR/+, open shapes) on control and lard diets. Data are pooled from six independent experiments. Log-rank, χ^2 = 112.6, p < 0.0001; Wilcoxon, χ^2 = 91.36, p < 0.0001 for flies on a lard diet.

(F and G) *upd3* and *Socs36E* transcription in flies as in (E) on lard or control diets for 30 days; three flies per sample; histogram represents mean ± SEM of four or five independent samples per diet. Please also see Figure S7.

42%; polyunsaturated, 9.5%; salt, nil; fiber, nil; Sainsbury's Basics Lard) in a weight-for-volume manner as described by Birse et al. (Birse et al., 2010), at either 6.3% or 15%. All experiments were carried out using the 15% lard-enriched diet except for experiments shown in Figures 3B and 3D, where a 6.3% lard-enriched diet was used.

Life Span Assays

Male flies were collected soon after eclosion, and groups of approximately 20–30 age-matched flies per genotype were placed on the given control and lipid-rich diets. All survival experiments were conducted at 25°C or 29°C. For temperature-sensitive plasmatocyte-depleted survival assays, fly crosses were carried out at 18°C to prevent Gal4 activity during development. Flies were collected soon after eclosion and were then placed at 29°C for 48 hr to initiate the inhibition of Gal80^{ts}, and therefore the transcription of *reaper*, to induce plasmatocyte cell death in adult flies. Once the plasmatocytes had been depleted in these flies, they were put in groups, per genotype, and placed on the given diets, and the survival assay was carried out at 25°C. In all other experiments utilizing Gal80^{ts}, crosses were also carried out at 18°C to prevent Gal4 activity during development. Flies were collected soon after eclosion over 2–3 days and were placed at 29°C for the entire survival assay, in order to ensure the overexpression or knockdown of *upd3* was maintained throughout the fly lifespan. In all survival assays carried out, vials were checked each day for any fly deaths, which were recorded. Food vials were kept on their sides to minimize the possibility of fly death from becoming stuck to the food, and vials were changed for fresh food every other day. Flies were transferred into new vials without using CO_2.

Plasmatocyte Deletion

Plasmatocyte cell death was triggered in adult flies by inducible expression of the proapoptotic protein *reaper* (*rpr*) (McGuire et al., 2004). *Rpr* expression was controlled by the temperature-sensitive driver *Tubulin*-Gal80^{ts} and the plasmatocyte-expressed driver *Hml*Δ-Gal4. In these flies the ubiquitous *Tub*-Gal80^{ts} promoter represses Gal4 activity in all tissues at temperatures below 29°C.

Insulin Sensitivity Experiment

Flies were injected with 50 nl of PBS or insulin-resuspended in PBS at a high dose (6.4 μg/ml) and a low dose (1.28 μg/ml). Flies from each condition were smashed in groups of three, in triplicate, 10 min postinjection in 75 μl of 2x Laemmli loading buffer (100 mM Tris [pH 6.8], 20% glycerol, 4% SDS, 0.2 M DTT). Samples were stored at −80°C until required. Eight microliters of this lysate was loaded per lane. Protein was transferred to nitrocellulose membranes. Antibodies used were as follows: from rabbit, anti-S505-phosphorylated *Drosophila* Akt (CST #4054, 1:1,000) and anti-pan-Akt (CST #4691, 1:1,000); from mouse, anti-α-tubulin (12G10 Developmental Studies Hybridoma Bank, used as an unpurified supernatant at 1:10,000). Secondary antibodies were HRP anti-rabbit IgG (CST #7074) and HRP anti-mouse IgG (KPL), both used at 1:5,000. Proteins were detected with Supersignal West Pico Chemiluminiscent Substrate (Thermo Scientific) using a BioRad Molecular Imager, and band densitometry was analyzed using Bio-Rad Image Lab software.

Glucose/Trehalose Measurement Assay

Flies were starved for 1 hr before being smashed in 75 μl TE + 0.1% Triton X-100 (groups of three flies per genotype and per diet were smashed per sample). After smashing, samples were immediately stored at −80°C. Samples were thawed when required and incubated at 90°C for 20 min to inactivate fly enzymes. Standards were prepared (ten series 1:2 dilution) using glucose for quantification. A total of 5 μl of sample was loaded per condition into flat-bottom 96-well tissue culture plates. Each fly sample was run three times, first alongside water for calculation of background fly absorbance, second with glucose reagent (Alere) for glucose, and third with glucose reagent plus trehalase for trehalose and glucose measurement. Plates were then incubated at 37°C for 1 hr before reading with a Wallac spectrophotometer at 490 nm.

Thin-Layer Chromatography Triglyceride Measurement

See Supplemental Experimental Procedures.

Feeding Assay

See Supplemental Experimental Procedures.

Quantitative Real-Time PCR

See Supplemental Experimental Procedures.

Confocal Microscopy

See Supplemental Experimental Procedures.

DiI-LDL Injection

See Supplemental Experimental Procedures.

Bacterial Infection

See Supplemental Experimental Procedures.

Fluorescence-Activated Cell Sorting

FACS samples were prepared as per Clark et al. (Clark et al., 2011). Approximately 90 adult male *Drosophila* (*w*;*Hml*Δ-Gal4,UAS-2xeGFP) were homogenized through a 70 μm cell strainer (BD Biosciences) with ice-cold 1x PBS-EDTA (2 mM) into a 50 ml falcon. The filtered sample was then centrifuged at 4°C for 15 min at 150 × *g*, the supernatant was discarded, and the pellet was resuspended in 5 ml of PBS-EDTA (2 mM) and passed once again through a 70 μm cell strainer; this wash step was repeated. GFP-positive *HmlΔ*-positive plasmatocytes were sorted through a 100 μm nozzle at 20 psi pressure using a FACS Aria II. For every sort, a GFP-negative sample was first analyzed in order to calibrate the sorter for detection of GFP-positive cells. Cells were sorted into RLT buffer from the RNeasy Plus Micro Kit (QIAGEN) in order to extract RNA for cDNA generation and perform RT qPCR, or cells were sorted into PBS in order to fix and stain them for imaging.

Oil Red O Staining of FACS-Sorted Plasmatocytes

See Supplemental Experimental Procedures.

Plasmatocyte Gene Expression Arrays

Plasmatocytes were isolated from *w*;*Hml*Δ-Gal4,UAS-2xeGFP flies that were either kept for 30 days on lard-supplemented food or normal food as described above. Five thousand to ten thousand GFP-positive plasmatocytes were FACS sorted from five independent samples per group and sorted directly into 6.4 μl preheated SuperAmp Lysis buffer (Miltenyi). Samples were incubated for 10 min at 42°C according to the manufacturer's guidelines and stored at −20°C until shipping. Samples were further analyzed and processed by the Miltenyi Genomic Service. For each of the cDNAs, 250 ng was used as template for Cy3 labeling according to the manufacturer's protocol. The Cy3-labeled cDNAs were finally hybridized to an Agilent Whole *Drosophila* Genome Oligo Microarrays Custom 8x60K. The Agilent Feature Extraction Software (FES) was used to read out and process the microarray image files. The software determines feature intensities (including background subtraction), rejects outliers, and calculates statistical confidences. Normalized data sets were compared using an unpaired t test ($p < 0.01$) and a fold change > 2 between normal diet and lard-supplemented food. Annotated significantly differentially regulated genes were depicted in a heat map. Heat map of differentially regulated genes represents the $\log_2$ of the fold change between normal diet and lard-supplemented diet.

Statistical Analysis

For real-time quantitative PCR, TLC and the colorimetric assay for glucose and trehalose levels standard sets were used in order to calculate values for each sample. An unpaired t test was used to calculate statistical significance for all experiments, excluding survival assays, where log-rank and Wilcoxon tests were performed using GraphPad Prism.

Transcription factor-binding site analysis on plasmatocyte microarray data was performed using Clover precisely as previously described (Clark et al., 2013). All genes up- or downregulated more than 2-fold and with $p < 0.01$ were used in this analysis. Further analysis of the plasmatocyte microarray data was done with Qlucore.

ACCESSION NUMBERS

MIAME-compliant raw data (gene expression profile of hemocytes from *Drosophila melanogaster* on lipid-enriched diet compared to normal diet) were deposited in the GEO database and can be accessed under the reference GSE63254.

SUPPLEMENTAL INFORMATION

Supplemental Information includes Supplemental Experimental Procedures and seven figures and can be found with this article online at http://dx.doi.org/10.1016/j.immuni.2014.12.023.

AUTHOR CONTRIBUTIONS

F.G. and K.J.W. designed the study and wrote the manuscript. K.J.W., K.K., C.A.P., and V.V. performed experiments. K.J.W, K.K., M.S.D., and F.G. designed experiments and analyzed the experimental data.

ACKNOWLEDGMENTS

Authors are indebted to Celine Trouillet and Marisol Gonzalez Melendez for help with cell sorting and maintaining the fly room; Dr. Claire Pean and Dr. Werner Wolfgang for help with experiments; Dr. Brian Stramer, Dr. Brigitte Senechal, and Andrew Page for critical reading of the manuscript and support; and Prof. Bruno Lemaitre, Dr. Nicolas Buchon, and Dr. James C.G. Hombria for helpful suggestions and the kind gift of *upd3* null, *mex1*-Gal4 flies, and UAS-upd2 flies. K.J.W. was a PhD fellow of the MRC (Medical Research Council, UK). C.A.P. is a fellow of the École de l'INSERM Liliane Bettencourt. K.K. was funded by a research fellowship by the DFG (German Research Foundation). This work was supported by a Wellcome Trust Senior Investigator award (WT101853MA) and ERC Investigator award (2010-StG-261299) from the European Research Council to F.G.

Received: December 17, 2013
Accepted: December 22, 2014
Published: January 15, 2015

REFERENCES

Agaisse, H., and Perrimon, N. (2004). The roles of JAK/STAT signaling in *Drosophila* immune responses. Immunol. Rev. *198*, 72–82.

Agaisse, H., Petersen, U.M., Boutros, M., Mathey-Prevot, B., and Perrimon, N. (2003). Signaling role of hemocytes in *Drosophila* JAK/STAT-dependent response to septic injury. Dev. Cell *5*, 441–450.

Al-Anzi, B., and Zinn, K. (2010). Colorimetric measurement of triglycerides cannot provide an accurate measure of stored fat content in *Drosophila*. PLoS ONE *5*, e12353.

Alikhani, N., Ferguson, R.D., Novosyadlyy, R., Gallagher, E.J., Scheinman, E.J., Yakar, S., and LeRoith, D. (2013). Mammary tumor growth and pulmonary metastasis are enhanced in a hyperlipidemic mouse model. Oncogene *32*, 961–967.

Arkan, M.C., Hevener, A.L., Greten, F.R., Maeda, S., Li, Z.W., Long, J.M., Wynshaw-Boris, A., Poli, G., Olefsky, J., and Karin, M. (2005). IKK-beta links inflammation to obesity-induced insulin resistance. Nat. Med. *11*, 191–198.

Bach, E.A., Ekas, L.A., Ayala-Camargo, A., Flaherty, M.S., Lee, H., Perrimon, N., and Baeg, G.H. (2007). GFP reporters detect the activation of the *Drosophila* JAK/STAT pathway in vivo. Gene Expr. Patterns *7*, 323–331.

Baranova, I.N., Kurlander, R., Bocharov, A.V., Vishnyakova, T.G., Chen, Z., Remaley, A.T., Csako, G., Patterson, A.P., and Eggerman, T.L. (2008). Role of human CD36 in bacterial recognition, phagocytosis, and pathogen-induced JNK-mediated signaling. J. Immunol. *181*, 7147–7156.

Betz, A., Ryoo, H.D., Steller, H., and Darnell, J.E., Jr. (2008). STAT92E is a positive regulator of *Drosophila* inhibitor of apoptosis 1 (DIAP/1) and protects against radiation-induced apoptosis. Proc. Natl. Acad. Sci. USA *105*, 13805–13810.

Binari, R., and Perrimon, N. (1994). Stripe-specific regulation of pair-rule genes by hopscotch, a putative Jak family tyrosine kinase in *Drosophila*. Genes Dev. *8*, 300–312.

Birse, R.T., Choi, J., Reardon, K., Rodriguez, J., Graham, S., Diop, S., Ocorr, K., Bodmer, R., and Oldham, S. (2010). High-fat-diet-induced obesity and heart dysfunction are regulated by the TOR pathway in *Drosophila*. Cell Metab. *12*, 533–544.

Biswas, S.K., and Mantovani, A. (2012). Orchestration of metabolism by macrophages. Cell Metab. *15*, 432–437.

Brown, S., Hu, N., and Hombría, J.C. (2001). Identification of the first invertebrate interleukin JAK/STAT receptor, the *Drosophila* gene domeless. Curr. Biol. *11*, 1700–1705.

Buchon, N., Broderick, N.A., Poidevin, M., Pradervand, S., and Lemaitre, B. (2009). *Drosophila* intestinal response to bacterial infection: activation of host defense and stem cell proliferation. Cell Host Microbe *5*, 200–211.

Charroux, B., and Royet, J. (2009). Elimination of plasmatocytes by targeted apoptosis reveals their role in multiple aspects of the *Drosophila* immune response. Proc. Natl. Acad. Sci. USA *106*, 9797–9802.

Clark, R.I., Woodcock, K.J., Geissmann, F., Trouillet, C., and Dionne, M.S. (2011). Multiple TGF-beta superfamily signals modulate the adult Drosophila immune response. Curr. Biol. *21*, 1672–1677.

Clark, R.I., Tan, S.W., Péan, C.B., Roostalu, U., Vivancos, V., Bronda, K., Pilátová, M., Fu, J., Walker, D.W., Berdeaux, R., et al. (2013). MEF2 is an in vivo immune-metabolic switch. Cell *155*, 435–447.

Clementi, A.H., Gaudy, A.M., van Rooijen, N., Pierce, R.H., and Mooney, R.A. (2009). Loss of Kupffer cells in diet-induced obesity is associated with increased hepatic steatosis, STAT3 signaling, and further decreases in insulin signaling. Biochim. Biophys. Acta *1792*, 1062–1072.

Dionne, M.S., Pham, L.N., Shirasu-Hiza, M., and Schneider, D.S. (2006). Akt and FOXO dysregulation contribute to infection-induced wasting in *Drosophila*. Curr. Biol. *16*, 1977–1985.

Driver, C.J., and Cosopodiotis, G. (1979). The effect of dietary fat on longevity of *Drosophila* melanogaster. Exp. Gerontol. *14*, 95–100.

Feng, B., Jiao, P., Nie, Y., Kim, T., Jun, D., van Rooijen, N., Yang, Z., and Xu, H. (2011). Clodronate liposomes improve metabolic profile and reduce visceral adipose macrophage content in diet-induced obese mice. PLoS ONE *6*, e24358.

Franc, N.C., Heitzler, P., Ezekowitz, R.A., and White, K. (1999). Requirement for croquemort in phagocytosis of apoptotic cells in *Drosophila*. Science *284*, 1991–1994.

Gold, K.S., and Brückner, K. (2014). *Drosophila* as a model for the two myeloid blood cell systems in vertebrates. Exp. Hematol. *42*, 717–727.

Goto, A., Kumagai, T., Kumagai, C., Hirose, J., Narita, H., Mori, H., Kadowaki, T., Beck, K., and Kitagawa, Y. (2001). A *Drosophila* haemocyte-specific protein, hemolectin, similar to human von Willebrand factor. Biochem. J. *359*, 99–108.

Han, M.S., Jung, D.Y., Morel, C., Lakhani, S.A., Kim, J.K., Flavell, R.A., and Davis, R.J. (2013). JNK expression by macrophages promotes obesity-induced insulin resistance and inflammation. Science *339*, 218–222.

Heinrichsen, E.T., and Haddad, G.G. (2012). Role of high-fat diet in stress response of *Drosophila*. PLoS ONE *7*, e42587.

Hirosumi, J., Tuncman, G., Chang, L., Görgün, C.Z., Uysal, K.T., Maeda, K., Karin, M., and Hotamisligil, G.S. (2002). A central role for JNK in obesity and insulin resistance. Nature *420*, 333–336.

Holz, A., Bossinger, B., Strasser, T., Janning, W., and Klapper, R. (2003). The two origins of hemocytes in *Drosophila*. Development *130*, 4955–4962.

Hou, X.S., Melnick, M.B., and Perrimon, N. (1996). Marelle acts downstream of the *Drosophila* HOP/JAK kinase and encodes a protein similar to the mammalian STATs. Cell *84*, 411–419.

Jamieson, A.M., Pasman, L., Yu, S., Gamradt, P., Homer, R.J., Decker, T., and Medzhitov, R. (2013). Role of tissue protection in lethal respiratory viral-bacterial coinfection. Science *340*, 1230–1234.

Jiang, H., Patel, P.H., Kohlmaier, A., Grenley, M.O., McEwen, D.G., and Edgar, B.A. (2009). Cytokine/Jak/Stat signaling mediates regeneration and homeostasis in the *Drosophila* midgut. Cell *137*, 1343–1355.

Jin, C., and Flavell, R.A. (2013). Innate sensors of pathogen and stress: linking inflammation to obesity. J. Allergy Clin. Immunol. *132*, 287–294.

Karpac, J., Younger, A., and Jasper, H. (2011). Dynamic coordination of innate immune signaling and insulin signaling regulates systemic responses to localized DNA damage. Dev. Cell *20*, 841–854.

Kohlmaier, A., and Edgar, B.A. (2008). Proliferative control in *Drosophila* stem cells. Curr. Opin. Cell Biol. *20*, 699–706.

Kroenke, C.H., Kwan, M.L., Sweeney, C., Castillo, A., and Caan, B.J. (2013). High- and low-fat dairy intake, recurrence, and mortality after breast cancer diagnosis. J. Natl. Cancer Inst. *105*, 616–623.

Krzemień, J., Dubois, L., Makki, R., Meister, M., Vincent, A., and Crozatier, M. (2007). Control of blood cell homeostasis in *Drosophila* larvae by the posterior signalling centre. Nature *446*, 325–328.

Krzemień, J., Oyallon, J., Crozatier, M., and Vincent, A. (2010). Hematopoietic progenitors and hemocyte lineages in the *Drosophila* lymph gland. Dev. Biol. *346*, 310–319.

Kurucz, E., Márkus, R., Zsámboki, J., Folkl-Medzihradszky, K., Darula, Z., Vilmos, P., Udvardy, A., Krausz, I., Lukacsovich, T., Gateff, E., et al. (2007). Nimrod, a putative phagocytosis receptor with EGF repeats in *Drosophila* plasmatocytes. Curr. Biol. *17*, 649–654.

Lebestky, T., Chang, T., Hartenstein, V., and Banerjee, U. (2000). Specification of *Drosophila* hematopoietic lineage by conserved transcription factors. Science *288*, 146–149.

Lemaitre, B., Nicolas, E., Michaut, L., Reichhart, J.M., and Hoffmann, J.A. (1996). The dorsoventral regulatory gene cassette spätzle/Toll/cactus controls the potent antifungal response in *Drosophila* adults. Cell *86*, 973–983.

Li, A.C., and Glass, C.K. (2002). The macrophage foam cell as a target for therapeutic intervention. Nat. Med. *8*, 1235–1242.

McGuire, S.E., Mao, Z., and Davis, R.L. (2004). Spatiotemporal gene expression targeting with the TARGET and gene-switch systems in *Drosophila*. Sci. STKE *2004*, pl6.

Medzhitov, R., Schneider, D.S., and Soares, M.P. (2012). Disease tolerance as a defense strategy. Science *335*, 936–941.

Meister, M. (2004). Blood cells of *Drosophila*: cell lineages and role in host defence. Curr. Opin. Immunol. *16*, 10–15.

Moore, K.J., and Tabas, I. (2011). Macrophages in the pathogenesis of atherosclerosis. Cell *145*, 341–355.

Olefsky, J.M. (2009). IKKepsilon: a bridge between obesity and inflammation. Cell *138*, 834–836.

Orr, J.S., Puglisi, M.J., Ellacott, K.L., Lumeng, C.N., Wasserman, D.H., and Hasty, A.H. (2012). Toll-like receptor 4 deficiency promotes the alternative activation of adipose tissue macrophages. Diabetes *61*, 2718–2727.

Osman, D., Buchon, N., Chakrabarti, S., Huang, Y.T., Su, W.C., Poidevin, M., Tsai, Y.C., and Lemaitre, B. (2012). Autocrine and paracrine unpaired signaling regulate intestinal stem cell maintenance and division. J. Cell Sci. *125*, 5944–5949.

Rahaman, S.O., Lennon, D.J., Febbraio, M., Podrez, E.A., Hazen, S.L., and Silverstein, R.L. (2006). A CD36-dependent signaling cascade is necessary for macrophage foam cell formation. Cell Metab. *4*, 211–221.

Rajan, A., and Perrimon, N. (2012). *Drosophila* cytokine unpaired 2 regulates physiological homeostasis by remotely controlling insulin secretion. Cell *151*, 123–137.

Reilly, J.J., and Kelly, J. (2011). Long-term impact of overweight and obesity in childhood and adolescence on morbidity and premature mortality in adulthood: systematic review. Int J Obes (Lond) *35*, 891–898.

Reilly, S.M., Chiang, S.H., Decker, S.J., Chang, L., Uhm, M., Larsen, M.J., Rubin, J.R., Mowers, J., White, N.M., Hochberg, I., et al. (2013). An inhibitor of the protein kinases TBK1 and IKK-ε improves obesity-related metabolic dysfunctions in mice. Nat. Med. *19*, 313–321.

Robertson, H.T., Smith, D.L., Pajewski, N.M., Weindruch, R.H., Garland, T., Jr., Argyropoulos, G., Bokov, A., and Allison, D.B. (2011). Can rodent longevity studies be both short and powerful? J. Gerontol. A Biol. Sci. Med. Sci. *66*, 279–286.

Saberi, M., Woods, N.B., de Luca, C., Schenk, S., Lu, J.C., Bandyopadhyay, G., Verma, I.M., and Olefsky, J.M. (2009). Hematopoietic cell-specific deletion of toll-like receptor 4 ameliorates hepatic and adipose tissue insulin resistance in high-fat-fed mice. Cell Metab. *10*, 419–429.

Singh, S.R., Liu, W., and Hou, S.X. (2007). The adult *Drosophila* malpighian tubules are maintained by multipotent stem cells. Cell Stem Cell *1*, 191–203.

Spann, N.J., Garmire, L.X., McDonald, J.G., Myers, D.S., Milne, S.B., Shibata, N., Reichart, D., Fox, J.N., Shaked, I., Heudobler, D., et al. (2012). Regulated accumulation of desmosterol integrates macrophage lipid metabolism and inflammatory responses. Cell *151*, 138–152.

Stuart, L.M., Deng, J., Silver, J.M., Takahashi, K., Tseng, A.A., Hennessy, E.J., Ezekowitz, R.A., and Moore, K.J. (2005). Response to Staphylococcus aureus requires CD36-mediated phagocytosis triggered by the COOH-terminal cytoplasmic domain. J. Cell Biol. *170*, 477–485.

Szendroedi, J., and Roden, M. (2009). Ectopic lipids and organ function. Curr. Opin. Lipidol. *20*, 50–56.

Tanti, J.F., Ceppo, F., Jager, J., and Berthou, F. (2012). Implication of inflammatory signaling pathways in obesity-induced insulin resistance. Front Endocrinol (Lausanne) *3*, 181.

van Herpen, N.A., and Schrauwen-Hinderling, V.B. (2008). Lipid accumulation in non-adipose tissue and lipotoxicity. Physiol. Behav. *94*, 231–241.

Wagener, A., Müller, U., and Brockmann, G.A. (2013). The age of attaining highest body weight correlates with lifespan in a genetically obese mouse model. Nutr Diabetes *3*, e62.

White, K., Tahaoglu, E., and Steller, H. (1996). Cell killing by the *Drosophila* gene reaper. Science *271*, 805–807.

Yan, R., Small, S., Desplan, C., Dearolf, C.R., and Darnell, J.E., Jr. (1996). Identification of a Stat gene that functions in *Drosophila* development. Cell *84*, 421–430.

Immunity, Volume *57*

Supplemental Information

Macrophage-Derived *upd3* Cytokine Causes Impaired Glucose Homeostasis and Reduced Lifespan in *Drosophila* Fed a Lipid-Rich Diet

Katie J. Woodcock, Katrin Kierdorf, Clara A. Pouchelon,
Valérie Vivancos, Marc S. Dionne, and Frédéric Geissmann

Supplemental information

Supplemental Figure Legends

Figure S1, related to Figure 1.

A. Survival of adult male Oregon-R flies fed a control diet or lard diet. Data pooled from 3 independent experiments. Log-rank χ^2=56.31, p<0.0001, Wilcoxon test χ^2=43.68, p<0.0001. **B.** Survival of w^{1118} flies fed a control or lard diet at 29°C. Data pooled from 6 independent experiments. Log-rank χ^2=212.6, p<0.0001, Wilcoxon χ^2=159.2, p<0.0001. **C.** Uptake of control and lard-enriched blue labelled food over 4 hours by groups of 10, 10 day old flies, n=4, mean absorbance at 625±SEM, p=0.652 ns. **D.** Time course of *Ilp2, 3* and *5* transcript levels in flies fed a control or lard diet at days 10 and 30, 3 flies per sample, histogram represents mean± SEM of 4-5 independent samples per diet and per time point. **E.** Repeat western blots as in Figure 1.D.

Figure S2, related to Figure 2.

A. Confocal microscopy of 10XSTAT92E-GFP reporter fly leg muscle after 10 days on a control or lard diet as a supplement to Figure 2.C. **B.** Confocal microscopy images of 10XSTAT92E-GFP reporter fly guts, flies were fed a control or lard diet for 20 days prior to dissection, as a supplement to Figure 2.D. **C.** *Defensin, Attacin-A, Metchnikowin, Cecropin A1* and *Drosocin* transcript levels in unchallenged (white bars) and *E. coli* and *M. luteus* infected flies (black bars) after 30 days of control or lard enriched diets. Histograms represent mean± SEM of 4-5 independent samples per diet.

Figure S3, related to Figure 3.

Representative confocal microscopy of $HmlΔ^+$ (white) and crq^+ (green) expression in a 10 day old fly. Bar: 50µm. Right: quantification *HmlΔ* and *crq* double positive and single positive cells, mean± SEM, n=4.

Figure S4, related to Figure 4.

A. Survival of inducible muscle (*24B*) driven *upd3* knock down flies (24BGal80^{ts}>*upd3*-IR, closed shapes) compared to control flies (UAS-*upd3*-IR/+, open shapes) on control diet and lard diet. Data pooled from 3 independent experiments. Log-rank χ^2=2.638, p=0.1043, Wilcoxon χ^2=0.3661, p=0.5452 for flies on a lard diet. **B.** Idem, for midgut (*mex1*) driven *upd3* knock down flies (*mex1>upd3*-IR, closed shapes) and control flies (UAS-*upd3*-IR/+, open shapes). Data pooled from 3 independent experiments. Log-rank χ^2=0.7216, p=0.3956, Wilcoxon χ^2=2.201, p=0.1379 for flies on a lard diet. **C.** Western blot analysis of p-AKT normalised to *Tubulin*, supplement to Figure 4D. **D-E.** Repeat western blots as in Figure 4D. **F.** Plasmatocyte numbers, ascertained by confocal microscopy of 65 day old *upd3* knock down (*Hml*Δ-Gal4,UAS-2xeGFP/*upd3*-IR, white bar) and control flies (*Hml*Δ-Gal4,UAS-2xeGFP/+, grey bar), mean± SEM, n=4.

Figure S5, related to Figure 5.

Survival of *upd3*-null rescue flies and controls fed a control diet. Data pooled from 5 independent experiments. Log-rank χ^2=44.40, p<0.0001, Wilcoxon χ^2=39.36, p<0.0001.

Figure S6, related to Figure 6.

A. Confocal images of plasmatocyte depleted and control flies injected with DiI-LDL, as a supplement to Figure 6. C. **B.** Transcript levels of *Ilp2*, *3* and *5* in plasmatocyte depleted and control flies fed a lard enriched diet for 20 days, 3 flies per sample, histogram represents mean± SEM of 4-5 independent samples per diet.

Figure S7, related to Figure 7.

A. Scavenger receptor screen summary table. Hemese, a glycophorin like membrane molecule (Kurucz et al., 2003), is a control expressed by plasmatocytes. Expression in sorted plasmatocytes and efficiency of plasmatocyte specific knock down (*HmlΔ*-Gal4) were assessed by qPCR. Knock down in the whole fly was studied using *Tub*-Gal4. **B.** Transcript level of *NimC1* and *crq* in plasmatocytes FACS sorted from *crq* and *NimC1* knock down flies and controls **C.** Survival of a second plasmatocyte-specific *crq* knock down fly line compared to controls. Data pooled from 4 independent experiments. Log-rank χ^2=114.9, p<0.0001, Wilcoxon χ^2=98.04, p<0.0001 for flies on a lard diet. **D.** Transcript levels of *Ilp2*, *3* and *5* in *crq*-IR/+ and

HmlΔ>crq-IR flies fed a lard enriched diet for 20 days, 3 flies per sample; histogram represents mean± SEM of 4-5 independent samples per diet. **E.** Survival of macrophage-specific *NimC1* knock down flies and controls. Data pooled from 3 independent experiments. Log-rank $\chi 2$=0.2991, p=0.5844, Wilcoxon $\chi 2$=0.1908, p=0.6623 for flies on a lard diet. **F.** Plasmatocyte numbers, ascertained by confocal microscopy of *bsk* knock down (white bar) and control flies (black bar), mean± SEM, n=4-5. **G.** *Socs36E* and *puckered (puc)* transcript levels in plasmatocyte *crq* knock down (*Hml*Δ>*crq*-IR, grey) and control (UAS-*crq*-IR/+, white) flies fed a control or lard diet for 30 days, 3 flies per sample, histogram represents mean± SEM of 4-5 independent samples per diet. **H.** Schematic model proposed to explain the roles of macrophages in the reduction of insulin sensitivity and lifespan due to lipid-rich diet.

Supplemental references

Hombria, J.C., Brown, S., Hader, S., and Zeidler, M.P. (2005). Characterisation of Upd2, a Drosophila JAK/STAT pathway ligand. Dev Biol 288, 420-433.

Kurucz, E., Zettervall, C.J., Sinka, R., Vilmos, P., Pivarcsi, A., Ekengren, S., Hegedus, Z., Ando, I., and Hultmark, D. (2003). Hemese, a hemocyte-specific transmembrane protein, affects the cellular immune response in Drosophila. Proc Natl Acad Sci U S A 100, 2622-2627.

Sinenko, S.A., and Mathey-Prevot, B. (2004). Increased expression of Drosophila tetraspanin, Tsp68C, suppresses the abnormal proliferation of ytr-deficient and Ras/Raf-activated hemocytes. Oncogene 23, 9120-9128.

Supplemental Experimental Procedures

***Drosophila melanogaster* stocks**

Fly stocks were maintained on food containing 10% w/v Brewer’s yeast, 8% fructose, 2% polenta and 0.8% Agar, at 25°C and 60% relative humidity. Male flies were used for all experiments.

**Fly stock**	**Description**
w^{1118};*Hml*ΔGal4,UAS-2xeGFP	Plasmatocyte specific reporter line, allowing for plasmatocyte visualisation (Sinenko and Mathey-Prevot, 2004). Gift of Sergey Sinenko.
w^{1118};;*Hml*Δ-DsRed	Plasmatocyte specific reporter line. Allowing for plasmatocyte visualisation in Gal4, UAS independent manner (Clark et al., 2011).
w^{1118};;*crq*-Gal4/TM6C.Sb^1	Plasmatocyte expressed driver.
w^{1118};UAS-2xeGFP;*crq*-Gal4/TM6C.Sb^1	Plasmatocyte expressed reporter line.
10X*STAT92E*-GFP flies	STAT-GFP reporter line (Bach et al., 2007)
w;;UAS-*reaper*/TM3	Overexpression line for proapoptotic protein reaper

	(*rpr*).
w^{1118};*Hml*ΔGal4,UAS-2xeGFP;Tub-Gal80^{ts}/TM6C.Sb^{1}	Temperature sensitive plasmatocyte specific reporter line.
wupd3 null (on the X chromosome)	Kindly donated by Bruno Lemaitre (Osman et al., 2012)
w;;UAS-*NimC1*-IR	Bloomington stock centre number: 25787
w;UAS-*crq*-IR & *w*;;UAS-crq-IR	VDRC stock numbers: 45883 (*crq*-RNAi-1) and 45884 (*crq*-RNAi-2)
w;UAS-*upd3*-IR	VDRC stock number: 106869
w;*upd3*-Gal4,UAS-GFP	Allowing for visualisation of $upd3^{+}$ cells
w;UAS-*Hemese*-IR	VDRC stock number: 107469
w;UAS-*CG10345*-IR	VDRC stock number: 100252
w;*mex1*-Gal4	Midgut specific driver line. Kindly donated by Nicolas Buchon.
w^{1118};UAS-*upd3*/SM6a	Line for over expression of *upd3*
w^{1118};;UAS-*upd3*/TM6C.Sb^{1}	Line for over expression of *upd3*
w;;UAS-*Myd88*-IR	VDRC stock number: 25399
w;UAS-*Imd*-IR	VDRC stock number: 101834
w;UAS-*Tak1*-IR	VDRC stock number: 101357
*w*UAS-*STAT92E*-IR;UAS-*STAT92E*-IR	Bloomington stock number: 26899 (on X) and VDRC stock number:106980 (on 2nd)
w;UAS-*bsk*-IR	VDRC stock number: 104569
w;UAS-*upd2*-GFP	Line for over expression of *upd2*. Kindly donated by James C G Hombria (Hombria et al., 2005)
w^{1118};;*Tubulin*-Gal4	Ubiquitous driver line
w^{1118};*Tubulin*-Gal80^{ts};*24B*-Gal4	Temperature sensitive muscle expressed driver line.
w;c564-Gal4;*Tubulin*-Gal80^{ts}	Temperature sensitive fat body specific driver line.

Quantitative Real-Time PCR

RNA extractions were carried out in TRIzol (Invitrogen) following the manufacturer's directions. cDNA synthesis was carried out using the First Strand cDNA Synthesis Kit (Thermo Scientific), priming with random hexamers (Thermo Scientific). PCR was performed with Sensimix SYBR Green no-ROX (Bioline) on a Corbett Rotor-Gene 6000. The cycling conditions used throughout were as follows: Hold 95°C for 10 minutes, then 40 cycles of 95°C for 15s, 63.4°C for 30s, 72°C for 30s. All calculated gene expression values were initially normalized to the value of the loading control gene, *Rpl1*, prior to further analysis.

The primer sequences used in this study are as follows:

Primer (qPCR)	Left	Right
Rpl1	TCCACCTTGAAGAAGGGCTA	TTGCGGATCTCCTCAGACTT
upd3	ACTGGGAGAACACCTGCAAT	GCCCGTTTGGTTCTGTAGAT
NimC1	CGCACCGAGTATTATACAGACG	TCCATGTTGAGGACACTGTTG
Ilp2	ATCCCGTGATTCCACCACAAG	GCGGTTCCGATATCGAGTTA

Ilp3	CAACGCAATGACCAAGAGAA	TGAGCATCTGAACCGAACT
Ilp5	GCCTTGATGGACATGCTGA	AGCTATCCAAATCCGCCA
Socs36E	AAAAAGCCAGCAAACCAAAA	AGGTGATGACCCATTGGAAG
crq	GCAGATAACCTTGTAGAGGATGG	CTCAGGTAAATGGGGATAGGTG
Dpt	ACCGCAGTACCCACTCAATC	CCCAAGTGCTGTCCATATCC
Drs	GTACTTGTTCGCCCTCTTCG	CTTGCACACACGACGACAG

Oil Red O staining of FACS-sorted plasmatocytes

See Supplemental Experimental Procedures.

FACS sorted plasmatocytes were stained for neutral triglyceride and lipid presence using Oil Red O (Sigma-Aldrich). Cells were sorted into 1x PBS-EDTA (2mM), after the sort cells were spun down at 4°C for 7 minutes at 150g. The cells were re-suspended in 50µl *Drosophila* Schneider's medium (Invitrogen), and were transferred onto a poly-L-lysine (Sigma-Aldrich) coated coverslip, which was placed inside a well of a 24 well non-tissue culture plate. After a 30 minute incubation at 37°C a further 500µl Schneider's medium was added and the incubation was repeated. All media was then removed and the cells were fixed with 4% PFA (paraformaldehyde) (Sigma-Aldrich) for 30 minutes at room temperature. After this the PFA was removed, and the cells were washed quickly with 60% isopropanol (Sigma-Aldrich). Oil Red O was then added to the cells for 30 minutes at room temperature. The Oil Red O was then removed and the coverslip was washed with distilled water. The coverslip was then inverted and mounted onto a superfrost slide (VWR International), in 10µl of Vectashield mounting media (Vector Laboratories) containing 1/1000 DAPI (4',6-diamidino-2-phenylindole) for nuclei staining. Mounting media was left to set for approximately 24 hours before cells were imaged with a confocal microscope (Leica SP5).

Thin layer chromatography (TLC) Triglyceride measurement

TLC experiments were adapted from (Al-Anzi and Zinn, 2010). Groups of 10 flies, in quadruplicate per genotype per diet, were anesthetised using CO_2 and then placed in 100µl of 3:1 ratio of chloroform: methanol. Samples were centrifuged for 3 minutes at 13,000rpm at 4°C, and then flies were smashed with pestles. A set of standards were prepared using lard in 3:1 chloroform;methanol for quantification. Samples and standards were loaded onto a silica gel, and a 4:1 mix of hexane: etheyl ether was prepared as the mobile phase. Gels were then stained using a general oxidising stain, ceric ammonium heptamolybdate (CAM), and baked at 80°C for 20 minutes. Baked gels were imaged with a BioRad Molecular Imager and triglyceride bands were quantified using BioRad Image Lab software.

Feeding assay

Blue diet food was prepared in the same way control or high fat diet, but 0.1% bromophenol blue and 0.5% xylene cyanol was added to the recipe. Feeding assay experiments were performed as described in (Rajan and Perrimon, 2012). Groups of 5 adult males (in triplicate or quadruplicate) were placed on blue food, and control non-

blue food for background subtraction for 4 hours at 25°C, flies were then decapitated to avoid the potential effects the eye pigmentation may have on absorbance values. The resulting fly torsos were then homogenised in PBS and centrifuged for 20 minutes. Supernatant was collected, and absorbance measured at 625nm.

Confocal microscopy
A Leica SP5 microscope was used for imaging, using either the 10x, NA (numerical aperture) 0.4 objective, the 20x Dry NA 0.5 objective, or the 40x Oil NA 1.25 objective. Images were acquired with a resolution of either 1024x1024 or 512x512, at a scan speed of 400Hz. Between 3 and 6 line averages were carried out and tile scanning was used in order to image whole flies. Flies were anaesthetised during imaging using a built in CO_2 chamber. FACS sorted, fixed and stained plasmatocytes were imaged through glass coverslips using the 40x Oil NA 1.25 objective. Images were processed using Fiji and Imaris 7.3.0. Cell counts and co-localisation counts (DiI-LDL$^+$ and *HmlΔ*$^+$ plasmatocytes) were performed using the MATLAB spot detection function in Imaris 7.3.0. Sum fluorescence intensity (SFI) values were obtained using Fiji, for Oil Red O stained cells SFI was normalised to cell size. In *upd3*-GFP flies SFI was measured in 3 size matched areas per fly.

Injection calibration (For DiI-LDL injection and infection assays)
Microinjection needles for fly injection were produced in the lab using glass capillaries and a needle puller. Injection was performed using a PicospritzerR III, and injection volume was calibrated by injecting a drop into a plot of oil. Expelled drops were measured to obtain a final injection volume of 50 nanolitres (nl). Flies were anesthetised with CO_2 under a light microscope on porous gas pads prior to abdominal injection.

Bacterial infection
Stocks of *Micrococcus luteus* (*M. luteus*) and *Escherichia coli* (*E. coli*) were stored at -80°C, cultures were grown from these stocks by selecting a small quantity of bacteria using a pipette tip and dipping the tip in LB broth. Individual *E. coli* and *M. luteus* cultures were grown in a 37°C shaking incubator overnight. The following morning cultures were centrifuged at 4°C at 1600rpm for 10 minutes. Bacterial pellets were re-suspended in PBS and the optical density (OD) was measured at the wavelength of 600nm (OD600). Mixed septic infections were carried out with an equal mix of OD-adjusted *E. coli* and *M. luteus* cultures, retaining a final OD of 1. Injection, and drop calibration was performed as described above on adult male flies aged between 7 and 10 days. Experiments also included PBS injected flies, as wounding controls and non-injected controls. After infection or wounding, flies were homogenised in TRIzol and stored at -20°C ready for cDNA synthesis and RT qPCR, in this case flies were homogenised 6 hours post infection in order to observe maximal AMP induction.

DiI-LDL injection
Adult male reporter flies aged between 7 and 10 days were injected, as described above, with 50nl of DiI-LDL (3,3'-dioctadecylindocarbocyanine-low density lipoprotein from human plasma, Sigma-Aldrich). Flies were imaged with a confocal microscope 1 hour (*HmlΔ*) or 24 hours (*upd3*-GFP) post injection for plasmatocyte uptake of the DiI-LDL.

Woodcock et al., Revised *SUPPLEMENTAL* Figure 1.

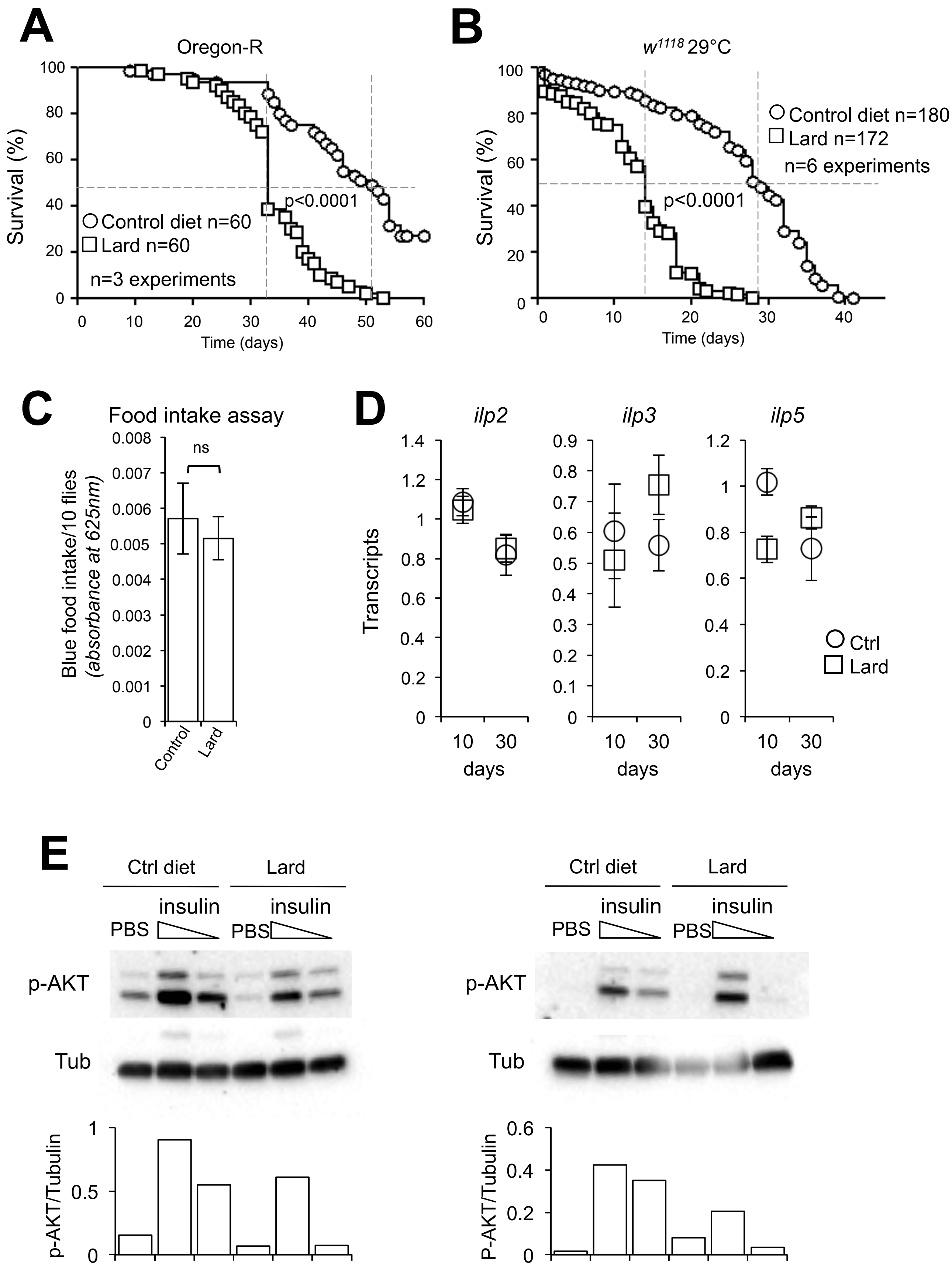

Woodcock et al., Revised *SUPPLEMENTAL* Figure 2.

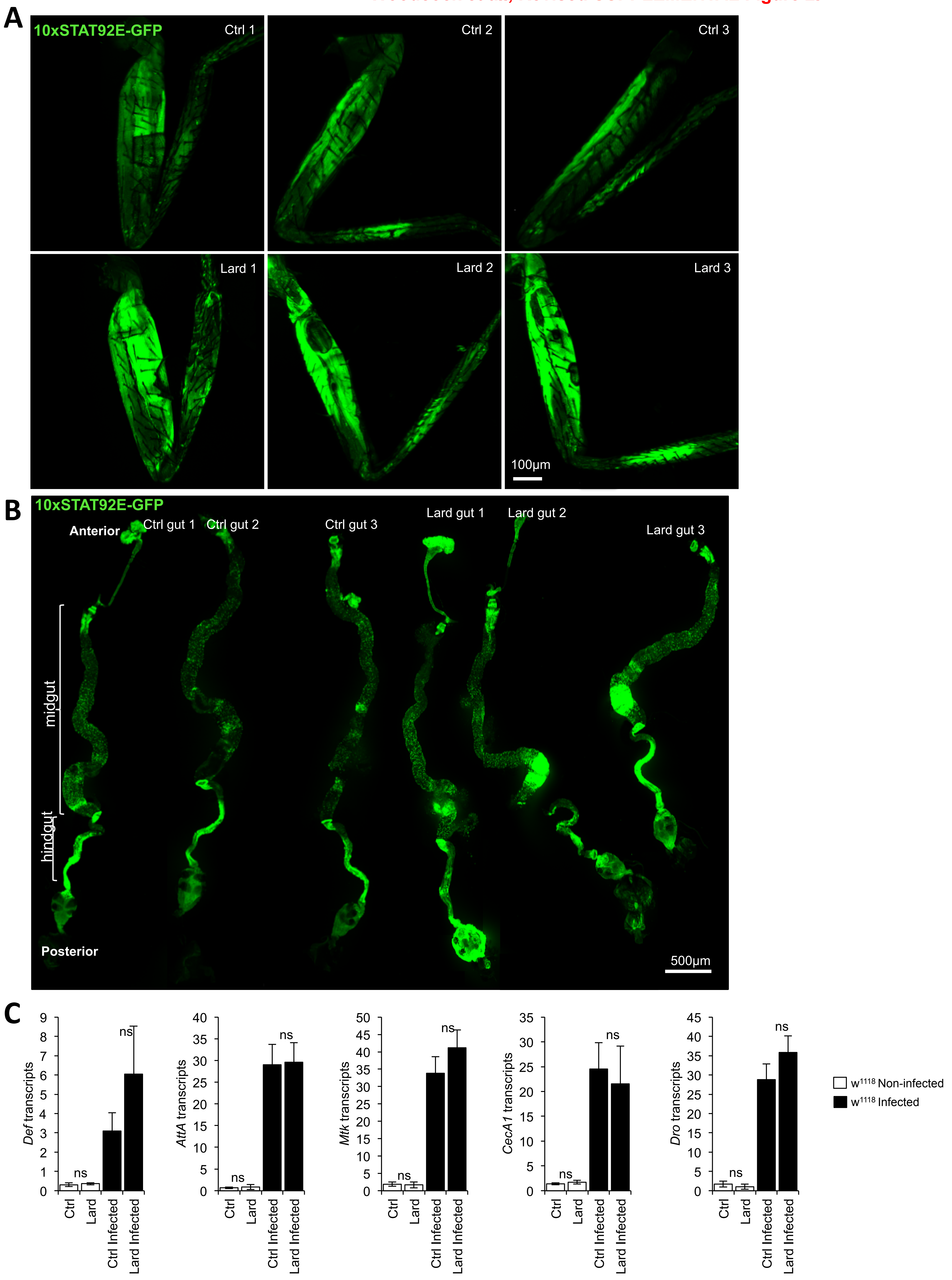

Woodcock et al., Revised *SUPPLEMENTAL* Figure 3.

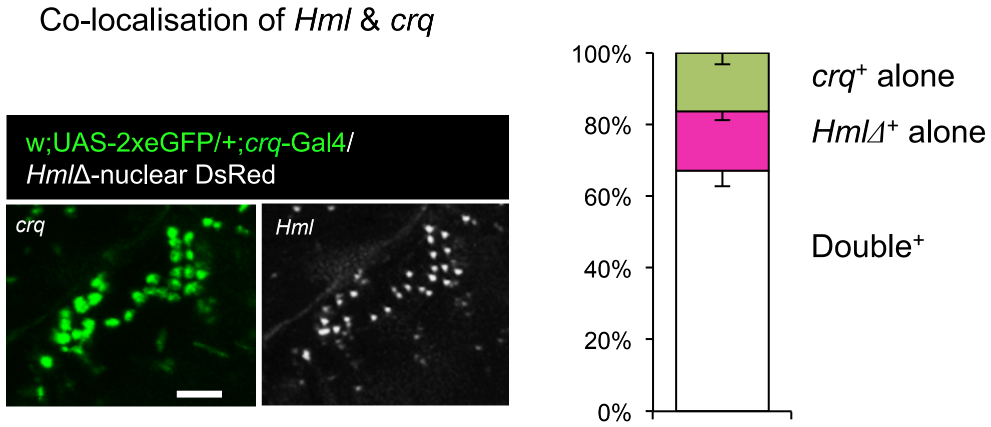

Woodcock et al., Revised *SUPPLEMENTAL* Figure 4.

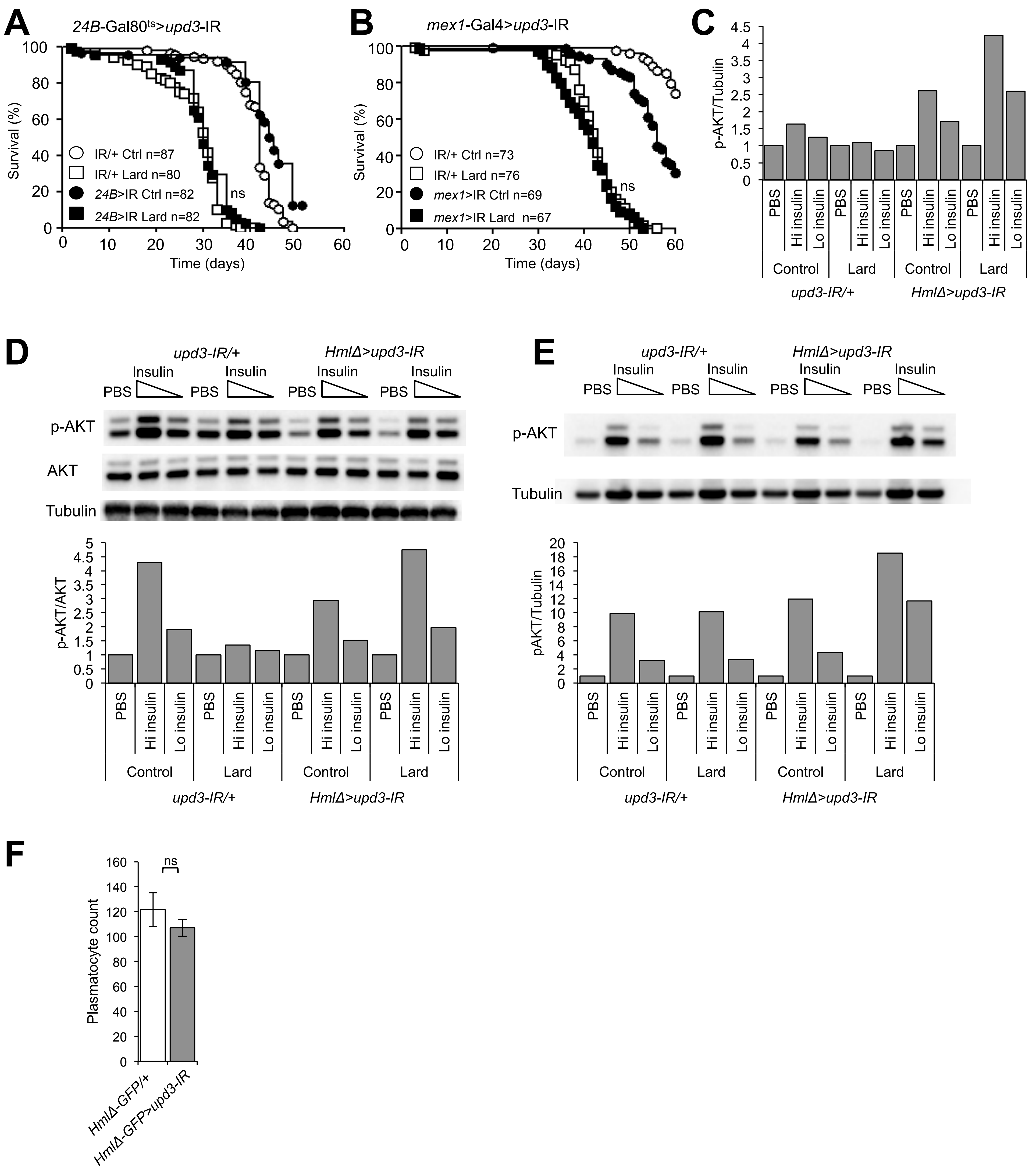

Woodcock et al., Revised *SUPPLEMENTAL* Figure 5.

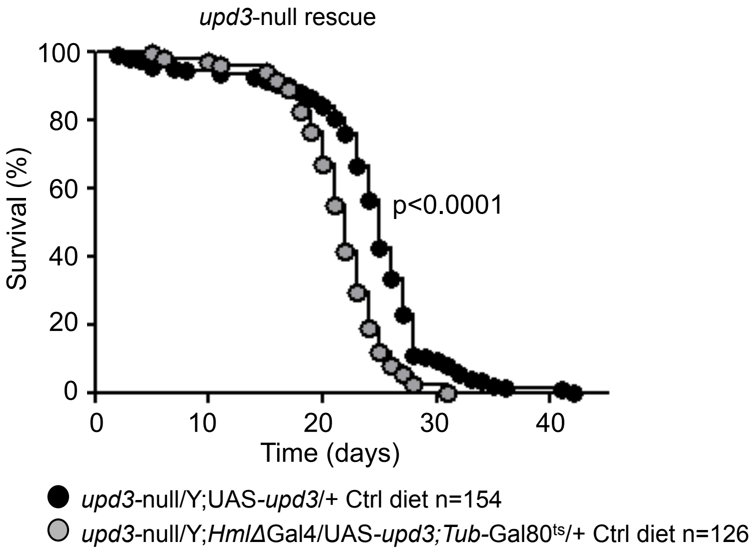

Log-rank X^2=44.40, P value P<0.0001
Wilcoxon X^2=39.36, P value P<0.0001

Woodcock et al., Revised *SUPPLEMENTAL* Figure 6.

A

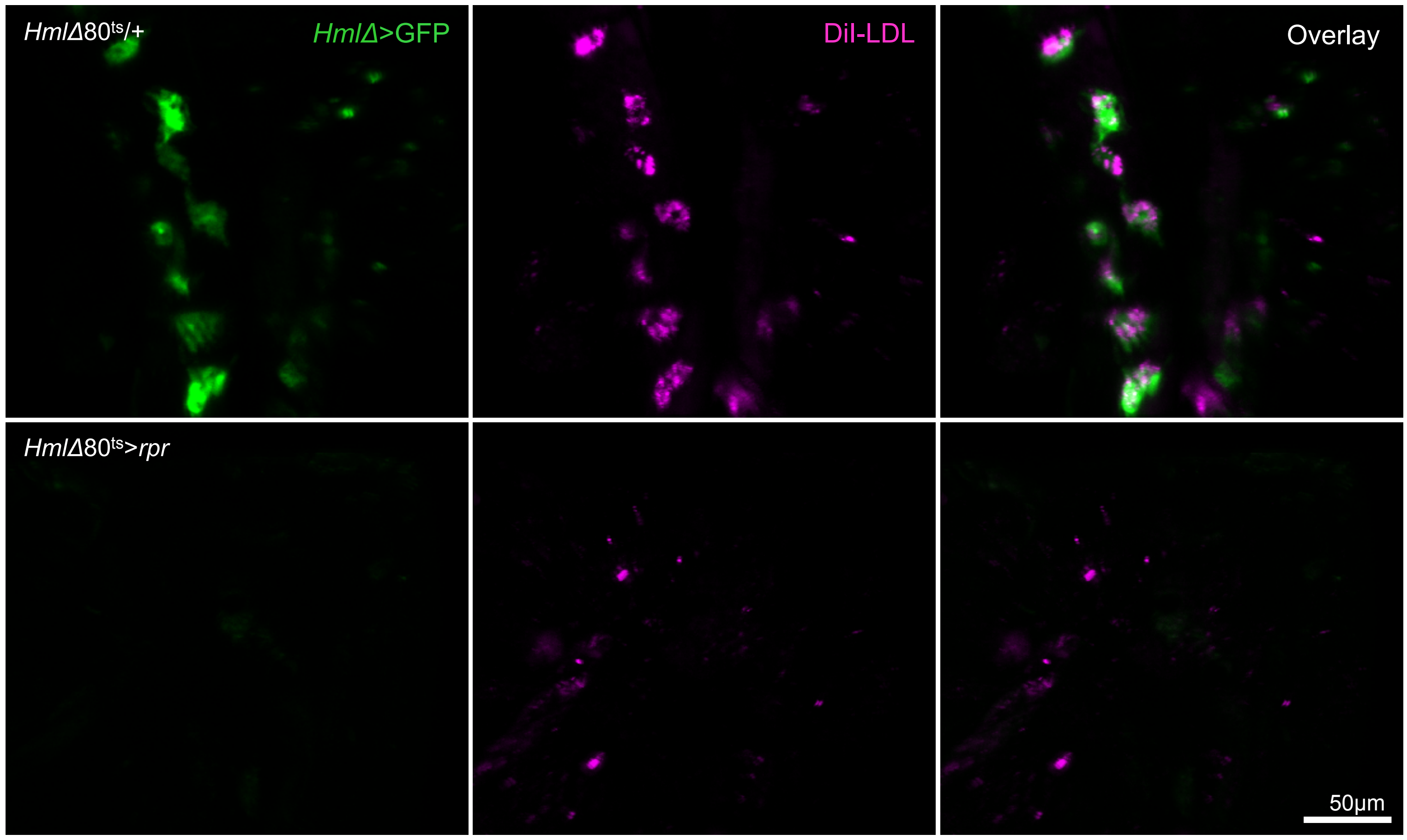

B

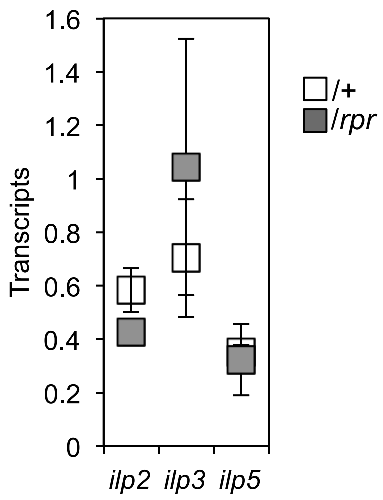

Woodcock et al., Revised *SUPPLEMENTAL* Figure 7.

A

Candidate gene	Expression in sorted hemocytes (qPCR)	Efficient knock down *HmlΔ*-Gal4	Efficient knock down with *Tub*-Gal4
Hemese	**+**	**+**	**+**
CG1887	No	Inconclusive	Not viable
CG2736	+	No	Not viable
NimC1	**+**	**+**	**+**
crq	**+**	**+**	**+**
CG3829	+	No	Not viable
CG7227	No	Inconclusive	Not viable
CG3212	No	No	No
peste	No	No	Not viable
CG10345	No	No	+
CG7422	No	Inconclusive	Not viable

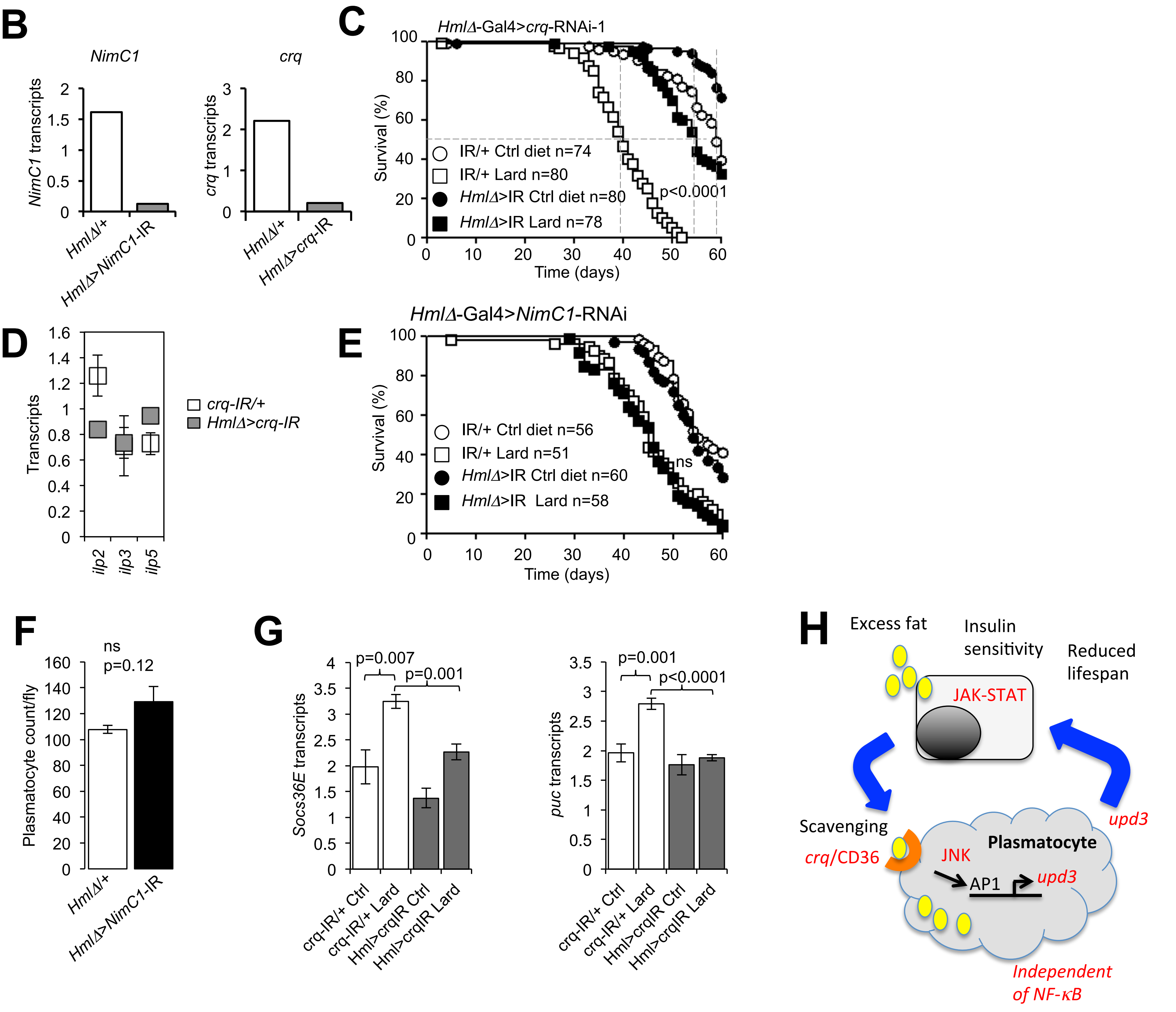

Supplement: Document S2. Article plus Supplemental Information [file mmc2.pdf]
